# Advances in Surface Biofunctionalization and Intelligent Monitoring of Vascular Scaffolds

**DOI:** 10.34133/research.1182

**Published:** 2026-03-11

**Authors:** Muhammad Rafique, Onaza Ali, Muhammad Shehr Yar Ali Khan Niazi, Jialing Zhang, Muhammad Shafiq, Jun Fang

**Affiliations:** ^1^ School of Biomedical Engineering and Med-X Research Institute, Shanghai Jiao Tong University, Shanghai 200240, China.; ^2^ Institute of Pediatrics, National Health Commission (NHC) Key Laboratory of Neonatal Diseases, Children’s Hospital of Fudan University, Shanghai 201102, China.; ^3^ Research Centre for Integrative Physiology & Pharmacology, Institute of Biomedicine, Faculty of Medicine, University of Turku, Turku, Finland.

## Abstract

Vascular scaffolds are fundamental devices in treating vascular occlusions, aneurysms, and hemodialysis access. However, their long-term efficacy is often compromised by 2 major pathophysiological responses: acute thrombosis and intimal hyperplasia, underscoring the need for effective antithrombotic treatment and intensive surveillance. This review highlights the emerging approaches used to address such challenges in vascular scaffolds: surface biofunctionalization and intelligent monitoring systems. We first introduce the leading biodegradable elastic polymers for vascular scaffolds, followed by a comprehensive overview of surface biofunctionalization techniques for preventing thrombosis and promoting endothelialization. The review further explores the cutting-edge advances in integrating flexible bioelectronics with cardiovascular implants for intelligent real-time monitoring of hemodynamics, thrombosis, and restenosis. It concludes with a discussion of the remaining challenges and future perspectives, thereby promoting the development of more effective cardiovascular therapies and their clinical applications.

## Introduction

Cardiovascular diseases (CVDs) are the leading cause of death and disability worldwide, with 19.2 million deaths and 437 million disability-adjusted life years (DALYs) in 2023. CVDs impose a substantial economic burden through direct healthcare costs and indirect losses from reduced workforce productivity. The growing prevalence of metabolic and lifestyle-related risk factors, combined with population aging, highlights the urgent need for effective prevention and intervention strategies [1–3]. The majority of CVDs arise from the narrowing or obstruction of vital blood vessels, leading to ischemia in affected organs. Common examples of CVDs include peripheral artery disease, aneurysmal diseases, cerebrovascular disease, deep vein thrombosis, and coronary artery disease [4].

Over the past few decades, various pharmacotherapies have been developed to modulate vascular tone, coagulation, and lipid accumulation in the vascular system to improve vascular function and patient quality-of-life (QOL). Current drug therapies include antiplatelet agents, β-blockers, angiotensin-converting enzyme (ACE) inhibitors, statins, and calcium channel blockers. Nevertheless, despite these advances in non-invasive treatments, the prevalence of CVDs continues to rise [5]. In the case of severe vascular occlusion or damage, surgical interventions are often required to restore blood flow. The interventions of angioplasty, percutaneous coronary intervention (PCI), and coronary artery bypass graft surgery (CABG) are selected based on the severity and complexity of the vascular occlusion [6]. Although PCI can mitigate various complications, including intimal hyperplasia (IH) formation through local delivery of anti-restenosis reagents, current drug-eluting stents (DES) often lack sufficient cell selectivity and sustained bioactivity. These limitations can result in late-stage in-stent restenosis (ISR) [7]. Vascular grafts are essential for reconstructing blood flow in advanced vascular disease with severe blockages and for creating arteriovenous (AV) access for hemodialysis [8,9]. While synthetic grafts made of polyethylene terephthalate (PET) or expanded polytetrafluoroethylene (ePTFE) are clinically effective for large-diameter vessels [10,11], they perform poorly in small-diameter applications (inner diameter < 6 mm), where they are prone to thrombosis, IH, and inadequate endothelialization [12–14]. To address these limitations, biodegradable vascular scaffolds made by synthetic and natural elastic materials have emerged as promising alternatives. These scaffolds offer excellent biocompatibility, tunable biodegradation, and mechanical compliance. Beyond providing mechanical support, they can mimic the native extracellular matrix (ECM) to facilitate vascular repair and regeneration. However, to achieve long-term patency, they still require surface modifications to resist thrombosis and suppress IH [15–18]. Looking beyond material-centric approaches, the integration of intelligent electronics represents a frontier strategy. This enables real-time, in situ monitoring of physiological conditions, facilitating early detection, timely therapeutic interventions, and improved patient outcomes.

**Fig. 1.**
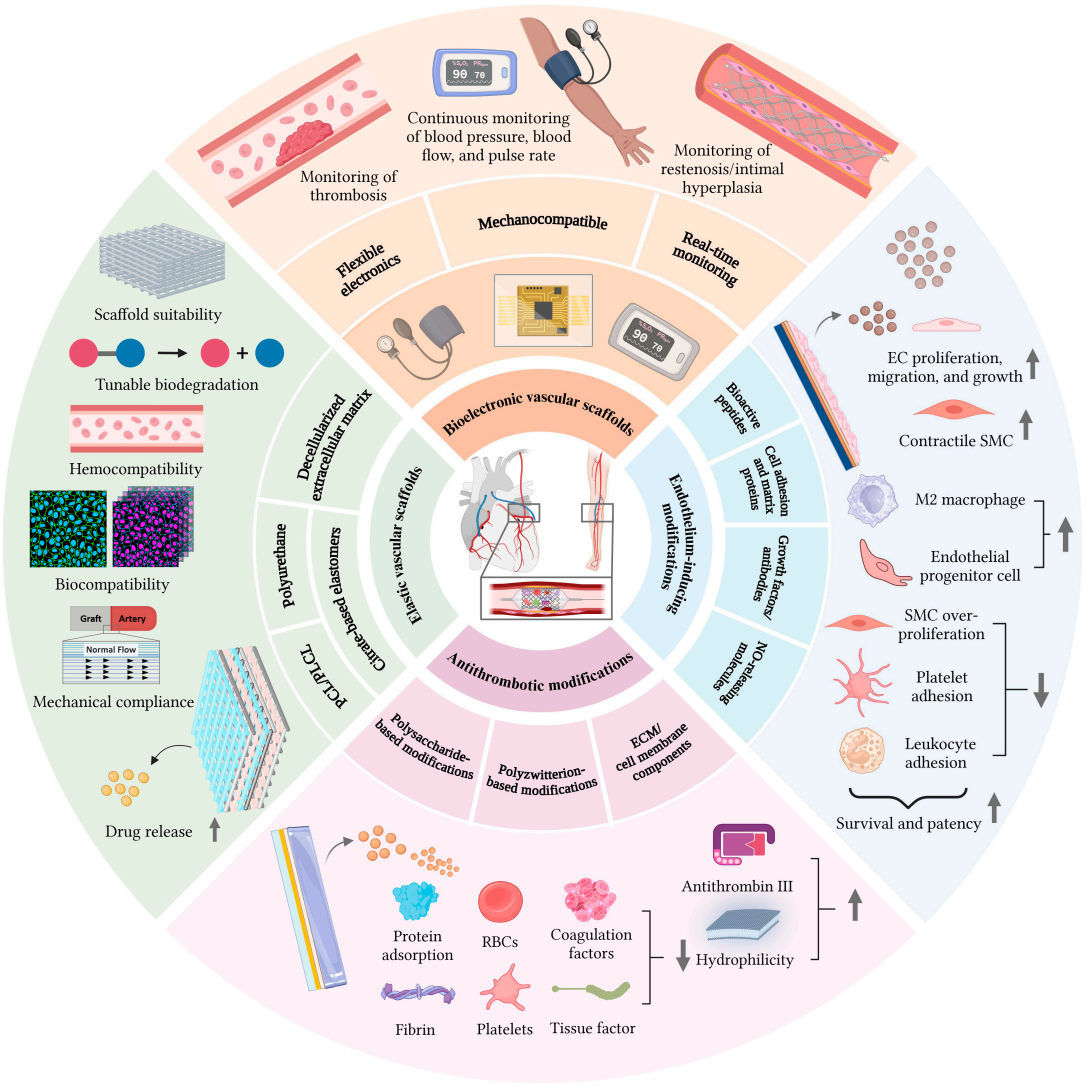
Schematic diagram illustrates various types of surface biofunctionalization strategies and intelligent monitoring of vascular scaffolds. Created with BioRender.com (https://BioRender.com/6biozr3).

Herein, we provide a comprehensive overview of the surface biofunctionalization of elastic vascular scaffolds, focusing on antithrombotic modifications (including polysaccharides, polyzwitterions, ECM, and cell membrane components) and endothelium-inducing strategies [including bioactive peptides, cell adhesion and matrix proteins, growth factors/antibodies, and nitric oxide (NO)-releasing molecules] (Fig. 1). Subsequently, we highlight the cutting-edge studies of intelligent vascular scaffolds that integrated with flexible electronics for real-time monitoring of hemodynamics, thrombosis, and restenosis. Finally, the challenges and future directions are discussed for guiding the development of next-generation multifunctional smart vascular scaffolds to support personalized theranostics and improved clinical outcomes. The review concludes with an outlook on the remaining challenges and future research directions, providing a roadmap for developing next-generation smart vascular scaffolds.

## Biodegradable Elastic Polymers for Vascular Scaffolds

The development of biocompatible and biodegradable biomaterials has fostered the generation of tissue-engineered constructs for regenerative medicine [19]. A wide range of synthetic, natural, and hybrid polymers have been extensively explored for the fabrication of vascular grafts and membrane covered stents. Synthetic polymers such as polycaprolactone (PCL), poly(glycolic acid) (PGA), poly(l-lactic acid) (PLLA), poly(l-lactic acid-co-*ε*-caprolactone) (PLCL), poly(lactic-co-glycolic acid) (PLGA), and polyurethane (PU) are widely used in biomedical applications owing to their tunable mechanical properties, controllable degradation, relatively low production costs, and ease of manufacturing [20]. In contrast, natural polymers such as collagen, elastin, silk, fibrin, and decellularized ECM (dECM) are promising alternative materials due to their inherent biodegradability, excellent biocompatibility, and low cytotoxicity [21]. Among diverse materials, synthetic and natural elastic materials have gained particular attention in the design and preparation of cardiovascular scaffolds. Unlike stiff substrates that can induce adverse physiological responses, such as chronic inflammation, fibrotic encapsulation, or disruption of native hemodynamic stresses, elastomer scaffolds can mitigate these risks by offering a dynamic, compliant interface that more harmoniously interacts with living tissue [22]. Importantly, vascular grafts based on biodegradable elastic polymers can match the mechanical characteristics of native arteries while offering superior suturability for surgical handling [23]. The following section provides a concise overview of several leading elastomers, including PCL, PLCL, PU, dECM materials, and citrate-based elastomers, highlighting their respective properties, advantages, and specific roles in cardiovascular scaffold design.

### PCL and PLCL

PCL is a versatile biodegradable polymer extensively used in vascular tissue engineering, particularly for small-diameter vascular grafts (SDVGs) [24]. It possesses good biocompatibility, suitable mechanical strength, slow biodegradation, and ease of processing and storage. Despite these mechanical advantages, PCL is intrinsically hydrophobic, which can limit cell adhesion and proliferation as well as increase the risk of platelet aggregation and IH. To overcome these limitations, various strategies have been employed, including blending with natural polymers and surface functionalization with bioactive molecules [25]. For example, vascular endothelial growth factor–class 1 hydrophobin (VEGF–HGFI)-modified electrospun PCL grafts displayed improved hemocompatibility through enhanced endothelial NO and prostacyclin (PGI₂) release. Upon in vivo implantation in the rat abdominal aorta, modified grafts demonstrated superior cellularization, endothelium formation, smooth muscle cell (SMC) regeneration, and blood capillary formation [26]. Similarly, polyphenol-based layer-by-layer (LBL) coatings using polyethyleneimine/epigallocatechin gallate–dexamethasone/heparin (PEI/EGCG-DEX/HEP) provided prolonged anticoagulant activity and anti-inflammatory effects, inhibited macrophage activation in vitro, and reduced inflammation in vivo [27]. A recent study employed carnosine–copper(II) functionalization, which simultaneously promoted NO generation to prevent thrombosis, reduced reactive oxygen species (ROS) through carnosine, and enhanced vascular remodeling. The modified grafts exhibited complete endothelial coverage, collagen deposition, and absence of thrombosis in a rat abdominal aorta model at 12 weeks post-implantation [28].

PLCL is a mechano-elastic, biocompatible, and biodegradable copolymer whose degradation rate and mechanical characteristics can be fine-tuned by adjusting the monomer ratio, molecular architecture, and degree of polymerization. The elasticity and compliance of PLCL copolymers closely resemble the mechanical properties of native blood vessels. Beyond its mechanical adaptability, PLCL can modulate cell behavior through mechanotransduction and promote endothelialization and vascular remodeling. However, similar to PCL, inherent hydrophobicity of PLCL copolymers limits the cytocompatibility, which necessitates bioactive modifications to enhance hydrophilicity and biocompatibility for vascular graft applications [29]. Kuang et al. [30] developed a PLCL-based core–shell nanofibrous graft using conjugate electrospinning and freeze-drying, in which the PLCL core imparted structural integrity, while a heparin/silk gel shell improved hemocompatibility and biocompatibility. This graft supported vascular regeneration and remained patent for over 8 months in vivo. Deng et al. [31] fabricated a fibrous SDVG with dual physical and biochemical cues by aligning nanogrooved PLCL microfibers and functionalizing the luminal surface of the graft with salvianolic acid B. This dual-functionalized graft design guided endothelial cell (EC) alignment and migration, which facilitated rapid endothelialization and inhibited thrombosis as well as improved SMC regeneration and collagen deposition without stenosis or thrombosis in a rat abdominal aorta model. Furthermore, a bioinspired multicomponent vascular graft was fabricated through a stepwise assembly combining melt electrowriting (MEW), lyophilization, and electrospinning, followed by heparin functionalization [32]. The MEW-fabricated PCL tubular frame mimicked the collagen–elastin architecture of the tunica media, while a fibrinogen-based lyophilized ECM promoted cell adhesion, infiltration, and endothelialization. An elastic outer layer based on electrospun PLCL fibers conferred additional compliance and mechanical reinforcement. This hybrid graft performed effectively as an abdominal aorta substitute in rats, displaying excellent hemocompatibility and mechanical properties comparable to native blood vessels.

### Polyurethane

PUs have been widely applied for numerous biomedical applications, including cardiovascular devices, heart valves, catheters, and artificial organs [33], because of their superior elastic properties, biocompatibility, and processability [34–36]. Particularly, PUs are considered as promising materials for making vascular scaffolds due to their superior mechanical compliance [37], while thrombosis and restenosis remain the major reasons leading to the failures of PU-based vascular scaffolds, thus warranting antithrombogenic surface treatment before surgical implantation [36,38]. Currently, a variety of strategies have been used for the biofunctionalization of PU-based vascular scaffolds. For instance, bilayered PU vascular grafts combining electrospinning and salt-leaching have been developed using methacrylated sulfated alginate to introduce heparin-like anticoagulant functionality. The resultant grafts possessed gradient porosity, improved adsorption of VEGF, reduced coagulation time, and facilitated endothelial monolayer formation in rat abdominal aorta implants for up to 5 months [39]. Similarly, the modification of PU with plasma ion immersion implantation (PIII) and tropoelastin coating enhanced its hydrophilicity and promoted cell adhesion and cytoskeletal organization, thereby improving anti-thrombogenicity and hemocompatibility [40]. In another report, polycarbonate urethane (PCU) scaffolds were treated with plasma to introduce amino (–NH_2_) groups; the latter facilitated heparin immobilization with varying surface density. The heparin modification of PCU reduced fibrin clot formation and improved patency of vascular scaffolds in a rat carotid artery implantation model [41].

However, traditional PUs usually lack reactive pendant groups for subsequent biofunctionalization to impart specific functionality for cell behaviors in tissue repair. Therefore, to address this challenge, our group and others have developed a series of novel biodegradable functional poly(ester urethane) urea (PEUU) elastomers with various reactive groups (including amino, carboxyl, cyclic disulfide, or their combinations) [42–45]. The developed polymers were fabricated into porous vascular scaffolds and further modified with antithrombotic or endothelium-inducing molecules (including zwitterionic compounds, heparin, and EC recruiting peptides), which demonstrated substantially improved hemocompatibility and pro-regenerative abilities [42,45].

### Decellularized ECM

The dECM preserves the native architecture and biochemical functions of tissues as it is prepared by removing the cellular components. The 3-dimensional (3D) porous structure of dECM provides an ideal scaffold for EC adhesion, proliferation, and migration. These properties make dECM a highly promising material for the fabrication of artificial blood vessels, thereby overcoming the limitations of synthetic materials, including poor durability, less patency, and mechanical instability. Although the application of dECM in SDVGs faces limitations in material availability and fixed lumen shapes, the development of membranous dECM and composite technologies has expanded its potential for size customization and clinical translation [46,47].

ECM scaffolds possess excellent biocompatibility and induce favorable immune responses. However, the lack of an hierarchical porous structure limits the directional cell migration and spatial organization, which hinders tissue integration. Zhu et al. [48] engineered ECM scaffolds by fabricating sacrificial PCL templates with aligned microfibers in membranous or tubular forms, followed by subcutaneous implantation in rats to induce cellularization and tissue formation, and finally removing the PCL template and cellular components to obtain dECM scaffolds with aligned microchannels**.** These scaffolds efficiently guided cell elongation, migration, proliferation, and maturation in vitro as well as promoted cellularization, vascularization, and favorable modulation of inflammatory responses in vivo across muscle, nerve, and artery models.

**Fig. 2.**
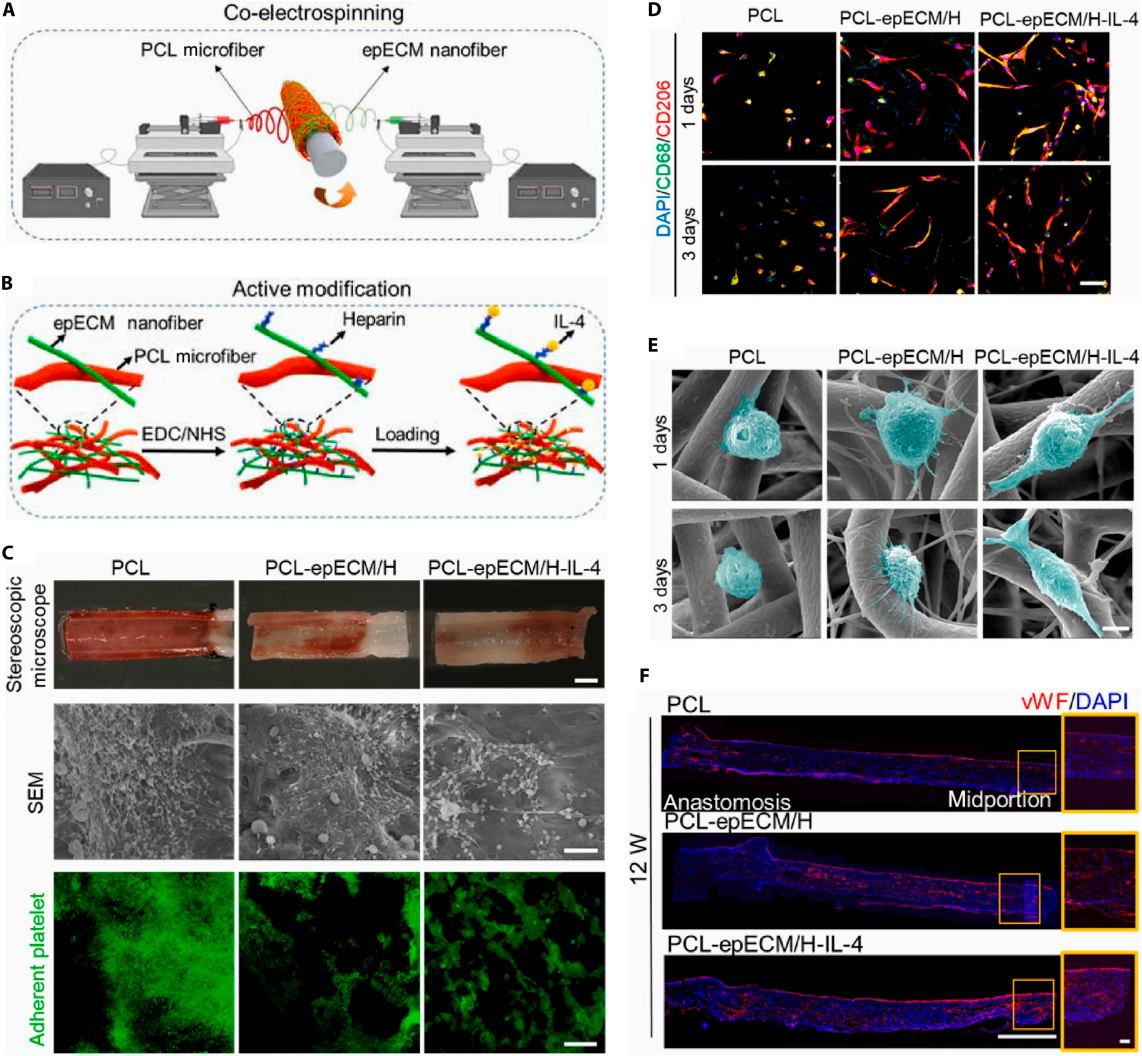
Effect of biofunctionalized hybrid vascular scaffolds on vascular regeneration. (A) Schematic illustration of the fabrication process of hybrid vascular grafts by co-electrospinning. (B) Schematic diagram shows an active modification process of the hybrid scaffolds. (C) Stereomicroscopy and scanning electron microscopy (SEM) images of the luminal surfaces of different grafts. Mepacrine staining shows platelet adhesion on the lumens of the grafts. (D) Double immunofluorescence (IF) staining images of nonpolarized macrophages (CD68, green) and anti-inflammatory macrophages (CD206, red) cultured on the scaffolds for up to 1 and 3 d. (E) SEM images showing the morphology of macrophages cultured on the scaffolds for up to 1 and 3 d. (F) IF images of longitudinal sections of the grafts stained for von Willibrand factor (vWF) (red) at 12 weeks. The orange box indicates the enlarged view of the graft wall. Cell nuclei are stained with 4′,6-diamidino-2-phenylindole (DAPI) (blue) [51]. Copyright 2022, KeAi.

Shi et al. [49] developed tri-layered vascular grafts integrating porcine thoracic aorta dECM powders with PLCL in the inner and middle layers and a PLCL outer sheath, alongside salidroside incorporated in the lumen. These grafts not only induced endothelial functions in vitro but also accelerated endothelialization, SMC regeneration, and ECM remodeling in vivo. Similarly, dECM/PLCL grafts functionalized with baicalin and a cathepsin S inhibitor prevented elastin degradation, reduced calcification, and induced M2 macrophage polarization, which ultimately enhanced vascular repair in a rat abdominal aorta model [50]. In a related study, to achieve immunomodulation and subsequent vascular regeneration, hybrid scaffolds composed of PCL microfibers and human placental ECM nanofibers were co-electrospun and further functionalized with heparin and interleukin-4 (IL-4) [51]. The modified vascular scaffolds demonstrated improved hemocompatibility and increased numbers of CD206^+^ macrophages, which displayed a spreading and elongated morphology as the culture time was increased. Additionally, the modified vascular grafts accelerated re-endothelialization and vascular remodeling in a rat abdominal aorta model for up to 12 weeks post-implantation (Fig. 2).

### Citrate-based elastomers

Citrate-based elastomers have attracted considerable attention for vascular scaffolds due to their intrinsic biocompatibility, mechanical tunability, and functional versatility. Citric acid, an intermediate of the tricarboxylic acid cycle, introduces multiple reactive carboxylic (–COOH) and hydroxyl (–OH) groups that enable controllable polymer crosslinking, biodegradation, and post-synthesis modification [52–54]. Particularly, poly(1,8-octanediol-co-citrate) (POC), synthesized via polycondensation of citric acid and 1,8-octanediol, exhibits elastomeric properties with endothelial compatibility [55,56]. Additionally, the functional groups of POC can be modified with different types of bioactive molecules [57].

**Fig. 3.**
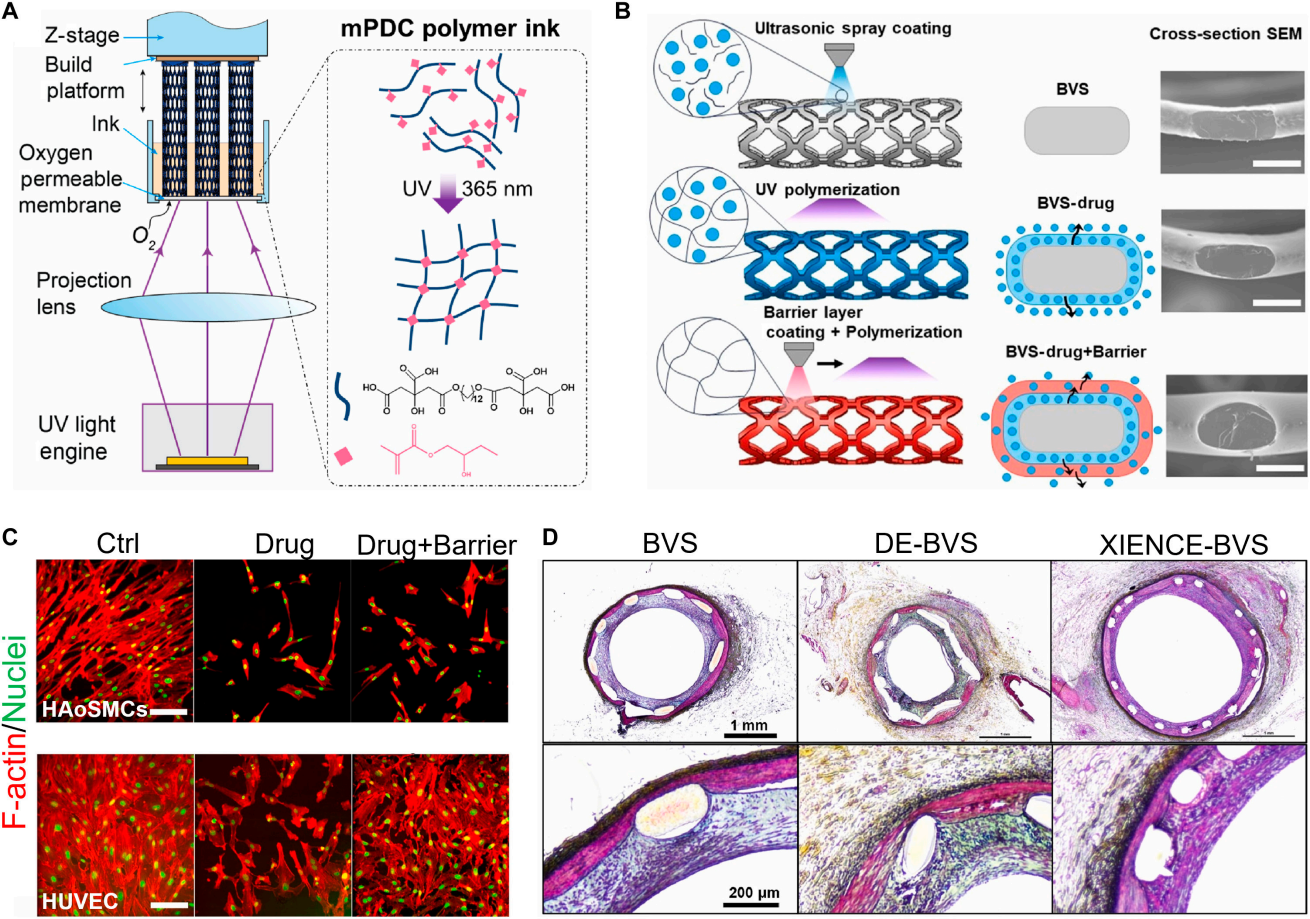
Effect of citrate-based 3D-printed bioresorbable vascular scaffold (BVS) on vascular regeneration and repair. (A) Schematic diagram illustrates the fabrication of 3D-printed BVS. (B) Illustration shows the procedure for the preparation the drug-coated BVS. SEM micrographs show cross-sections of various BVS groups. (C) IF staining images of F-actin and nuclei in human aortic smooth muscle cells (HASMCs) and human umbilical vein endothelial cells (HUVECs) grown on different types of BVSs. (D) Movat pentachrome staining images of swine coronary arteries with implanted scaffolds/stents at 28 d post-implantation [62]. Copyright 2024, KeAi.

Kibbe and colleagues [58] used POC as a surface coating for ePTFE vascular grafts. POC-coated vascular grafts demonstrated biocompatibility, hemocompatibility, and stability for up to 28 d in vitro. In vivo implantation of POC-coated vascular grafts revealed enhanced endothelialization and patency in rat carotid arteries. Similarly, ePTFE grafts were cross-linked with poly(diol citrate) (PDC) elastomers, followed by modification with diazeniumdiolation for in situ NO production [59]. When used as perivascular wraps in rat carotid injury models, these NO-releasing PDC coatings inhibited IH and maintained mechanical compliance comparable to native blood vessels [60]. In a similar study, in situ light-activated vascular scaffolds (ILVSs) were developed using methacrylate PDC (mPDC) and chitosan-based NO donors. ILVSs indicated durable compression strength for 7 months and they continuously released NO at least for up to 3 h, and they were able to be coated onto porcine arteries ex vivo [61]. Ding et al. [62] employed mPDC as a 3D-printable bioink and produced dual-layer drug-eluting bioresorbable vascular scaffolds (BVSs). The inner methacrylate POC (mPOC)–drug layer and outer pure mPOC layer modulated the release kinetics of everolimus, enabling 60% and 80% drug release at days 7 and 14, respectively, compared with 92% drug release without the barrier. Additionally, these scaffolds facilitated human umbilical vein endothelial cell (HUVEC) growth, inhibited the proliferation of human aortic smooth muscle cells (HASMCs), and reduced ISR in porcine coronary arteries at day 28 post-surgery (Fig. 3).

Vascular oxidative stress drives the phenotypic transition of vascular SMCs to osteochondral-like cells and thus promotes calcification and restenosis [63,64]. To mitigate this, ascorbic acid (AA) has been incorporated into POC networks, yielding poly(1,8-octanediol-co-citrate-co-ascorbate) (POCA) [65]. The POCA-coated grafts suppressed vascular smooth muscle cell (VSMC) proliferation, inhibited lipid peroxidation, and reduced IH in guinea pig aortic models [66]. Furthermore, vitamin A-derived all-trans retinoic acid (atRA) is known to regulate cell proliferation and differentiation and has been locally immobilized on POC membranes to avoid systemic toxicity [67,68]. The atRA-loaded POC coatings provided sustained release, reduced macrophage infiltration, and decreased IH by 56% in a rat carotid artery implantation model [69]. Similarly, dual-layer atRA-based coatings improved retention, enhanced endothelialization, and prevented restenosis [70]. Moreover, the direct incorporation of atRA into the polymer backbone produced poly(1,8-octamethylene-citrate-co-retinate) (POCR), which improved cytocompatibility, reduced the migration and proliferation of HASMCs, and provided intrinsic antioxidative activity [71].

Taken together, various elastomers have been explored for vascular scaffolds (Table 1). Synthetic materials offer advantages in mechanical properties, cost, and ease of manufacturing, while natural materials provide superior bioactivity. Notably, all vascular scaffolds need to be modified to prevent thrombosis and promote rapid endothelialization.

**Table 1.** Elastomers for vascular repair: Composition, modification, and performance in preclinical models

Type of polymer	Fabrication method	Type of modification	Animal model	Results	Ref.
PCL	Electrospinning	Fusion protein VEGF–HGFI	Rat abdominal aorta—1 month	Improved hemocompatibility, enhanced endothelialization, capillary formation, and SMC regeneration	[26]
	Electrospinning	PEI/EGCG-DEX/HEPARIN	Rat subcutaneous implantation model—15 and 30 d; rabbit AV shunt model—1 h	Sustained anticoagulant activity and anti-inflammatory effect, inhibited macrophage activation, improved hemocompatibility	[27]
	Electrospinning	Carnosine–Cu(II)	Rat abdominal aorta—12 weeks	Catalytic NO generation prevented thrombosis, reduced ROS, and achieved complete endothelial coverage and collagen deposition	[28]
PLCL	Electrospinning and freeze-drying, PLCL as core and heparin/silk as shell	Heparin/silk gel	Rabbit carotid artery—2, 4, and 8 months	Improved hemocompatibility and biocompatibility, supported vascular regeneration, graft patent >8 months	[30]
	Electrospinning	Salvianolic acid B	Rat abdominal aorta—4 weeks	Guided EC alignment and migration for rapid endothelialization, inhibited thrombosis, improved SMC regeneration and collagen deposition	[31]
PU	Electrospinning and salt-leaching	Methacrylated sulfated alginate	Rat abdominal aorta—1 and 5 months	Gradient porosity enhanced VEGF adsorption, reduced coagulation time, promoted endothelialization and long-term patency	[39]
	Electrospinning	Heparin	Rat carotid artery—2 and 4 weeks	Enhanced anti-thrombogenic activity in vitro, improved patency at 2–4 weeks, endothelial monolayer formed at 4 weeks	[41]
dECM-based	Electrospinning, PLCL/dECM inner and middle layers, PLCL outer layer	Salidroside	Rat abdominal aorta—3 and 6 weeks	dECM layers improved endothelialization, SMC regeneration, and ECM deposition, salidroside inhibited thrombosis and improved patency	[49]
	Co-electrospinning PLCL/dECM	Baicalin and cathepsin S inhibitor	Rat abdominal aorta—3 months	Enhanced endothelialization, prevented elastin degradation and calcification, induced M2 macrophage polarization	[50]
	Co-electrospinning PCL/dECM	Heparin and IL-4	Rat abdominal aorta—12 weeks	Reduced thrombosis, increased anti-inflammatory macrophages and endothelialization, promoted SMC regeneration and ECM deposition	[51]
Citrate-based	3D printing	Everolimus	Swine coronary artery—28 d	Controlled drug release suppressed HASMC proliferation, promoted HUVEC growth, and reduced in-stent restenosis	[62]
	Coating on synthetic ePTFE graft	Ascorbic acid	Guinea pig aorta—4 weeks	Suppressed SMC proliferation, exhibited iron chelation and antioxidant activity in vitro, inhibited intimal hyperplasia in vivo	[66]

PCL, polycaprolactone; VEGF, vascular endothelial growth factor; SMC, smooth muscle cell; PEI, polyethyleneimine; EGCG, epigallocatechin gallate; DEX, dexamethasone; AV, arteriovenous; NO, nitric oxide; ROS, reactive oxygen species; PLCL, poly(l-lactide-co-*ε*-caprolactone); EC, endothelial cell; PU, polyurethane; 3D, three-dimensional; HUVEC, human umbilical vein endothelial cell; HASMC, human aortic smooth muscle cell; ePTFE, expanded polytetrafluoroethylene; dECM, decellularized extracellular matrix; IL-4, interleukin-4

## Antithrombotic Modifications for Vascular Scaffolds

The interaction of the vascular devices and blood components can initiate thrombosis formation at the blood–material interface, which can limit the effectiveness of these devices [72]. One of the limiting factors leading to thrombosis and foreign body reactions of vascular devices is the lack of inherent antithrombotic surface properties [73]. Antithrombotic surfaces play a crucial role in minimizing thrombotic risk as they effectively prevent thrombogenesis without compromising hemostasis [74]. Currently, various antithrombotic modifications based on polysaccharides, polyzwitterions, ECM, and cell membrane components have been exploited to improve the thromboresistance and biocompatibility of vascular scaffolds.

### Polysaccharide-based modifications

Heparin is a natural glycosaminoglycan [75], which binds and induces conformational changes in anti-thrombin, leading to the inhibition of thrombin and factor Xa (FXa), thereby producing effective antithrombotic effects [76]. Heparin-based modifications have been widely explored to enhance the hemocompatibility of vascular devices [77,78]. In addition to the covalent immobilization of heparin with vascular devices, it can be physically incorporated into bioactive modifications via LBL self-assembly method owing to its anionic nature. For example, vascular tubes based on ε-polylysine (ε-PL) and sodium alginate were coated with heparin and laminin-derived Tyr-Ile-Gly-Ser-Arg (YIGSR) peptide using LBL self-assembly. Since heparin was directly coated on vascular tubes containing cationic ε-PL, it exhibited rapid release, followed by a sustained release of YIGSR, which considerably improved the hemocompatibility of vascular tubes as well as induced endothelium formation in vitro. This strategy supports the fabrication of coaxially printed vascular grafts using a biologically inspired release system, which possesses the potential to improve the patency of SDVGs [79]. Heparin grafting on electrospun PEUU–gelatin vascular grafts has been shown to substantially enhance hemocompatibility and biocompatibility [80]. In vitro, heparin-modified grafts effectively reduced platelet adhesion while promoting EC adhesion and proliferation. Furthermore, when implanted as abdominal aorta substitutes in rats for up to 1 and 4 weeks, heparinized grafts promoted rapid endothelialization and facilitated the infiltration and growth of SMCs within the graft wall.

The LBL self-assembly method can also be used to incorporate bioactive components into modifications, such as polyphenols in conjunction with other bioactive cues, including EGCG, copper ions, and heparin [81]. For instance, PLLA-based vascular stents were LBL coated with EGCG/Cu^2+^ and heparin; while EGCG can stabilize the coating through various intermolecular interactions and extend the release of heparin for up to 90 d, Cu^2+^ can catalyze NO production from endogenous S-nitrosothiols (RSNO), thereby simultaneously reducing thrombosis, resolving inflammation, and promoting EC adhesion and growth. More importantly, these coatings regulated the inflammatory response by suppressing pro-inflammatory cytokine production in macrophages in vitro and inhibiting SMC proliferation, thereby reducing IH formation in a rabbit abdominal aorta model at 3 months post-implantation.

Fucoidan, a sulfated glycan from brown algae, has substantial anticoagulant potential [82]. Fucoidan administration was shown to reduce the adhesion of platelets and leukocytes, suppress the proliferation of SMCs, inhibit thrombin formation, and inactivate free radical formation [83]. Yim and colleagues [84] have developed fucoidan-based modifications and successfully used them to improve endothelium induction and the hemocompatibility of polyvinyl alcohol (PVA)-based vascular grafts. Briefly, PVA was mixed with fucoidan and crosslinked with sodium metaphosphate, which enabled robust vascular grafts with desired mechanical properties. Besides, fucoidan-based modifications can be exploited in conjunction with other biophysical cues, e.g., topography. While fucoidan can suppress the formation of focal adhesion kinase and promote the attachment and growth of ECs, topographical modulation can promote the migration of ECs, thereby improving the hemocompatibility of the vascular device compared to that of the ECM-based modifications or bare vascular grafts. Plane PVA and modified PVA groups can effectively reduce platelet adhesion and thrombin generation as compared to the collagen-coated group. Similarly, fucoidan-immobilized and topographically-modulated vascular grafts have been shown to enhance in situ endothelialization and improve the patency rate of vascular grafts in a rabbit carotid artery implantation model [85].

To enhance the adhesion of ECs, a polysaccharide-based composite hydrogel coating was developed using an inner layer of dopamine-modified hyaluronic acid (HA) containing heparin and an outer gelatin layer containing chondroitin sulfate (CS). The coating was shown to release heparin in a slow and sustained manner, where approximately 65% of the loaded heparin was released over 96 h due to the cross-linked HA layer, while CS was almost completely released within 24 h owing to the rapid degradation of gelatin. It was demonstrated that the coating can improve blood compatibility and promote adhesion and migration of ECs in electrospun PCL fibers [86].

### Polyzwitterion-based modifications

Polyzwitterionic polymers, particularly polybetaines such as poly(2-methacryloyloxyethyl phosphorylcholine) (PMPC), sulfobetaine (SB), and carboxybetaine (CB), are widely used as antithrombotic surface modifications for vascular scaffolds and other blood-contacting devices. These materials contain both cationic and anionic groups within each repeat unit, which promote strong electrostatically induced hydration and form a dense water barrier at the material surface. This hydration layer effectively suppresses nonspecific protein adsorption, especially fibrinogen, and thereby prevents subsequent platelet adhesion, activation, and aggregation that initiate thrombus formation. In addition, phosphorylcholine (PC)-based polymers closely mimic zwitterionic phospholipid moieties of cell membranes, which further enhance hemocompatibility and reduce thrombogenic responses at the blood–material interface. Consequently, polyzwitterionic modifications have been shown to considerably improve the thromboresistance and hemocompatibility of vascular scaffolds under blood-contacting conditions [87–89].

#### Phosphorylcholine

PC-based polyzwitterionic modification is an effective strategy to improve the hemocompatibility of blood-contacting devices and materials, which can also be tailored by incorporating additional moieties to regulate cell adhesion, proliferation, and migration. For instance, one group developed a PC-based polyzwitterionic coating on PLLA stents by copolymerizing 2-methacryloyloxyethyl phosphorylcholine (MPC), 2-methylene-1,3-dioxepane (MDO), and butyl methacrylate (BMA) [90]. Each component exhibits a distinct function: The MDO provides degradability, MPC imparts antifouling properties, and BMA promotes coating adhesion. The resulting modification can be enzymatically degraded by lipase, enabling controlled release of rapamycin to suppress the ISR. Notably, in a porcine coronary artery injury model, this PC-based modification not only inhibited IH but also substantially reduced levels of IL-6, monocyte chemoattractant protein-1, and collagen deposition at 42 d post-implantation.

Decellularized tissues and tissue-engineered vascular grafts represent a highly promising strategy for creating SDVGs. The decellularization approach recapitulates the hierarchical architecture of native tissues while preserving many beneficial components for promoting tissue repair and regeneration [46,91,92]. However, decellularization also leads to the exposure of many pro-thrombotic ECM components, such as collagen fibers. To address this problem, PMPC has been applied to modify decellularized bovine intercostal arteries and demonstrated to improve the biocompatibility, mechanical stability, and anticoagulation performance. In a rabbit carotid artery replacement model, all unmodified grafts were blocked within 1 week, while PMPC-modified grafts showed good patency and notable SMC layer reconstruction after 30 d post-implantation [93].

As compared to the surface modification of vascular devices, bulk modification of the polymer used to fabricate a device may be more advantageous since it can incorporate pendant reactive groups for the subsequent functionalization with bioactive moieties. In a previous study, PEUU polymers with pendant amino (–NH₂) groups were synthesized, which were then utilized to incorporate carboxylated PC to suppress platelet adhesion and modulate SMC growth in a dose-dependent manner in vitro [42]. Similarly, PEUU containing disulfide bonds and pendent NH₂ groups was synthesized, which enabled co-functionalization with heparin and endothelium-inducing peptide (TPS: TPSLEQRTVYAK) [45]. While heparin was conjugated to the NH₂ groups through amide bond formation, TPS-maleimide was conjugated with PEUU using click chemistry [94]. This dual modification of PEUU suppressed platelet adhesion while promoting endothelial progenitor cell (EPC) adhesion and proliferation in vitro. These functional PEUU-based platforms may be utilized to incorporate multiple bioactive cues for synergistic antithrombotic effect and endothelium induction.

#### Poly(sulfobetaine)

**Fig. 4.**
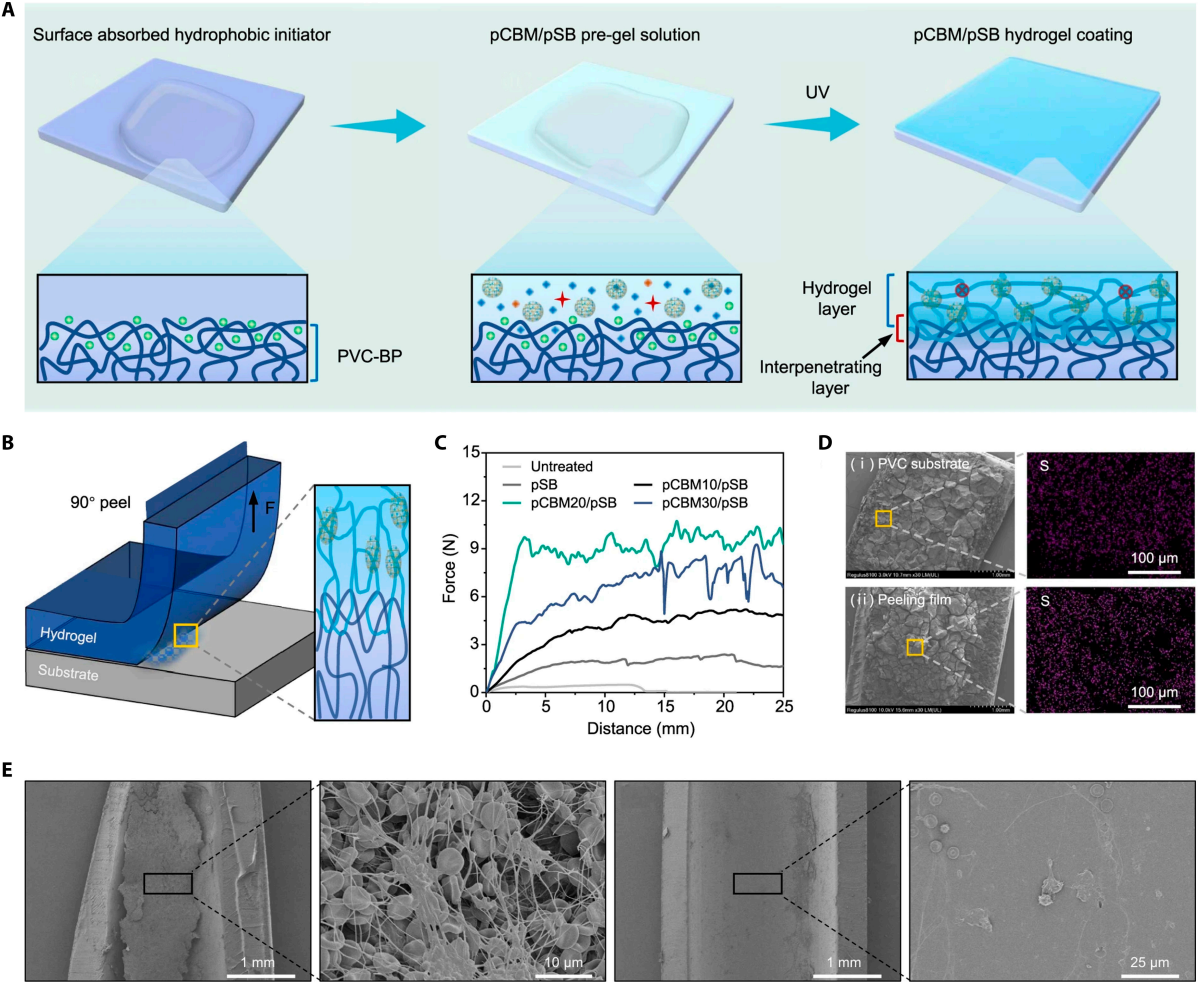
Preparation, adhesion stability, and antithrombotic performance of poly(sulfobetaine)-based modification on blood-contacting substrates. (A) Schematic illustration for the preparation of the pCBM/pSB hydrogel coating. (B) Schematic diagram of the peeling test. (C) Force–displacement curves. (D) SEM images and sulfur element mapping demonstrate coating adhesion and retention on both the PVC substrate and the peeling film. (E) SEM images show extensive thrombus formation on unmodified PVC tubing, while the pCBM/pSB-coated tubing shows markedly reduced thrombus accumulation after 2 h of blood circulation [96]. Copyright 2022, Springer Nature.

Since vascular devices can fail due to thrombosis, which can be aggravated by plasma protein adsorption as well as coagulation pathway and complement system activation, luminal modifications of the device with zwitterionic polymers may obviate these limitations [95]. For instance, a zwitterionic hydrogel coating was developed by reinforcing a poly(sulfobetaine) (pSB) matrix with poly(carboxybetaine) microgels (pCBMs), which enhanced the mechanical strength and resistance to swelling of the pSB. The coating was developed by pretreating polyvinyl chloride (PVC) with a hydrophobic photoinitiator, followed by coating with pCBM/pSB pre-gel, whereby methacrylated SB monomers (SBMA) were diffused into the PVC interface. Upon ultraviolet (UV) irradiation, SBMA can be polymerized to form a pCBM/pSB hydrogel coating through entanglement with PVC chains (Fig. 4A). The coating was firmly bound to the PVC surface (Fig. 4B), and an increasing concentration of pCBM further enhanced the adhesion strength of the coating (Fig. 4C). Moreover, the coating remained visible on both the PVC surface and peeling film even after the peeling test (Fig. 4D), and it effectively prevented thrombus formation in a rabbit model of extracorporeal circulation (Fig. 4E) [96].

Polyelectrolytes possess both cationic and anionic groups, which can provide a flexible and stable coating ability on various substrates. Wang et al. [97] prepared a bilayer hydrogel coating by exploiting the flexibility and adhesiveness of polyelectrolytes and antifouling properties of polyzwitterions. The coating was prepared via a 2-step dip-coating method using a bottom adhesive hydrogel layer (PEI and acrylic acid) applied to various substrates, followed by UV-mediated polymerization of SB and SBMA in situ. To further enable an integration between the top and bottom layers, double bonds were introduced into the bottom layer using N-succinimidyl acrylate. This functional modification suppressed platelet adhesion and protein adsorption as well as resisted thrombotic production due to enhanced surface hydrophilicity, lubricity, and anti-biofouling ability. However, cytocompatibility and in vivo biocompatibility of these modifications yet remain to be evaluated.

In an independent study, SBMA was introduced to PU substrates using a linker-free PIII method and shown to increase hydrophilicity, and reduce fibrinogen adsorption and thrombus deposition compared to the uncoated PU [98]. While zwitterions can impart anti-fouling characteristics to vascular devices, they can impede the adhesion of desirable vascular cells onto the lumen surface in vitro and in vivo. To address this challenge, Yao et al. [99] exploited dihydrolipoic acid-functionalized SB-derived starch-based hydrogel coating to modify PET. The PET was modified with polydopamine (PDA), followed by the immobilization of starch-based coating via a Michael addition reaction between the catechol groups of PDA and dihydrolipoic acid groups of functionalized starches. In addition, the REDV (Arg-Glu-Asp-Val) peptide was introduced into modifications, which facilitated the adhesion of HUVECs. This dual modification of PET with SB and cell-adhesive peptide improved hemocompatibility (e.g., reduced protein adsorption and suppressed platelet adhesion), diminished inflammatory response, and promoted favorable cell adhesion and phenotype (e.g., enhanced vascular cell adhesion, proliferation, and migration of HUVECs, while less adhesion and proliferation of SMCs)*.*

#### Poly(carboxybetaine)

CB, a zwitterionic derivative of betaine structurally analogous to SB, differs primarily in its anionic group, where a carboxylate moiety replaces the sulfonate group. This structural variation results in a lower charge density difference between the cationic and anionic groups of CB, leading to weaker intramolecular associations and superior hydration in aqueous environments [88]. Leveraging these hydration and antifouling characteristics, CB-based zwitterionic modifications have emerged as effective surface-engineering strategies to improve the hemocompatibility and biofunctionality of vascular scaffolds. Yang and colleagues [100] developed a mussel-inspired zwitterionic coating to functionalize polylactic acid (PLA) stents. They synthesized a poly(carboxybetaine acrylate-co-dopamine methacrylate) (PCBDA) copolymer and immobilized it onto a PDA–PEI pretreated stent surface via catechol-mediated crosslinking, forming a robust PCBDA/PDA–PEI interface. The zwitterionic PCBDA layer established a dense hydration shell that effectively suppressed nonspecific protein adsorption and platelet activation, resulting in superior anticoagulant and anti-inflammatory performance. Particularly, while inhibiting SMC proliferation, the coating selectively supported EC adhesion and growth due to the adhesive catechol functionalities. In vivo implantation of stents demonstrated pronounced inhibition of IH and inflammatory responses.

Wang et al. [101] designed a hydrogen sulfide (H₂S)-releasing and CB-modified SDVG that combined hemocompatibility with regenerative functionality. The graft was fabricated by co-electrospinning PCL with a keratin-based H₂S donor, followed by surface modification with zwitterionic CB via dopamine-assisted self-polymerization. This dual-functional design ensured controlled H₂S release, enhanced blood compatibility, increased resistance to the oxidative stress, and enhanced EC adhesion and migration alongside reduced SMC proliferation. Under dynamic perfusion conditions, the graft facilitated endothelial monolayer formation. Further, in vivo studies in a rat carotid artery model confirmed accelerated endothelialization and reduced calcification.

In particular, endothelium-promoting strategies can be coupled with antithrombotic polyzwitterion modifications to achieve the synergistic performance. For example, one study coated the glass slides using a CB-based copolymer poly(CBMA-r-BMA) and EC-specific peptide "REDV", which yielded excellent hemocompatibility, as characterized by minimal platelet adhesion and activation, and preferential EC proliferation while suppressed SMC attachment and growth [102]. Similar phenomena were also observed by modifying the titanium implants with REDV-conjugated poly(CB)-based hydrogel [103]. Thus, integrating anti-fouling polyzwitterion modification with EC-capturing peptide conjugation on the cardiovascular implants may provide a promising approach to prevent thrombosis, promote in situ endothelialization, and ultimately improve long-term therapeutic outcomes.

### ECM component-based modifications

**Fig. 5.**
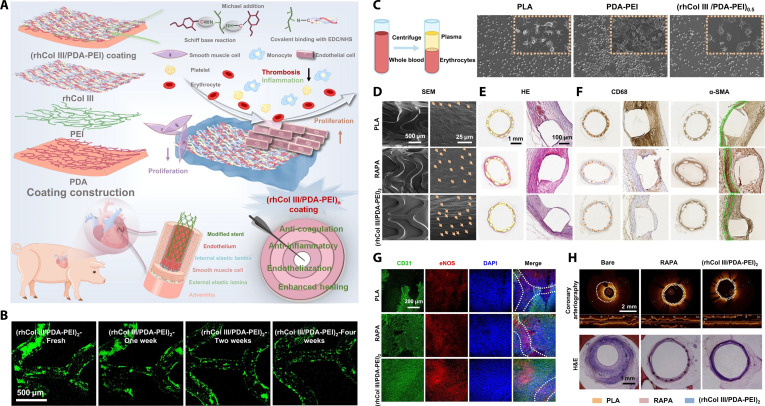
Anticoagulant and endothelium-promoting vascular stent modification based on an ECM protein. (A) Schematic of PLA stent coating based on recombinant humanized collagen type III (rhCol III) and its multiple functions. (B) Stability of (rhCol III/PDA–PEI)_2_ coating after balloon dilation under the simulated flowing system with bovine blood serum. (C) SEM images of platelet adhesion on uncoated, PDA–PEI-coated, and (rhCol III/PDA–PEI)n-coated PLA sheets. (D) SEM micrographs of the luminal surface of bare, RAPA-modified, and (rhCol III/PDA–PEI)_2_-modified PLA stents. (E to G) Histological evaluation of stented rabbit abdominal aortae 3 months post-implantation: (E) hematoxylin and eosin (H&E) staining, (F) IHC staining of CD68 and α-smooth muscle actin (α-SMA), and (G) IF staining of CD31 and eNOS. (H) Optical coherence tomography and H&E staining of porcine coronary arteries 3 months after stent deployment [107]. Copyright 2024, Springer Nature.

Collagen is a major component of the ECM that not only maintains the mechanical elasticity of tissues but also provides structural support for cellular activities. While animal-derived collagens have been widely used in tissue engineering and regeneration medicine [104,105], their thrombogenic properties limit their suitability for blood-contacting cardiovascular devices. To address this, a recombinant humanized type III collagen (rhCol III) was developed, which is composed of 16 tandem repeats of the triple-helix T16WTp fragment. This peptide is derived from the Gly^483^-Pro^512^ sequence of human type III collagen, and it contains the cell adhesive motifs Gly–Glu–Arg (GER) and Gly–Glu–Lys (GEK) while excluding the platelet-binding hydroxyproline (O). Compared with animal-sourced collagen, PLA substrates coated with a stepwise assembly of (HA/rhCol III)_6_ exhibited reduced platelet adhesion and activation, promoted EC proliferation, and suppressed SMC proliferation. In a rabbit abdominal aorta model, compared to bare PLA and rapamycin-eluting PLA stents (RAPA), the (HA/rhCol III)_6_-coated PLA stents induced less inflammatory response and IH, along with enhanced endothelialization at 3 months post-implantation [106]. Further work by the same group produced a more stable, covalent conjugated (rhCol III/PDA–PEI)_2_ coating on PLA stents (Fig. 5A and B). This modification also reduced platelet adhesion, promoted rapid endothelialization, and induced less inflammatory response and IH in a rabbit abdominal aorta model (Fig. 5C to G). It also diminished neointimal thickening in porcine coronary artery models at 3 months post-implantation (Fig. 5H) [107]. In addition, the rhCol III can be directly loaded into vascular grafts to improve hemocompatibility and increase EC adhesion and growth. Bilayered electrospun vascular grafts, composed of an inner layer of 60% rhCol III-loaded PLCL and an outer layer of PLCL, also exhibited reduced platelet adhesion and increased EC adhesion compared to bare PLCL grafts. When implanted as femoral artery substitutes in beagle dogs, these grafts improved EC regeneration at 3 months post-implantation [108]. Collectively, recombinant humanized collagen represents a marked advance in cardiovascular biomaterials, enabling the design of hemocompatible, endothelial-friendly implants that mitigate thrombosis and restenosis. By combining enhanced bioactivity with reduced immunogenicity, rhCol III-based modifications may offer a promising, drug-free strategy for improving the long-term performance of diverse blood-contacting cardiovascular devices and scaffolds.

HA, as one of the main components of the ECM, mediates various cellular activities, whereby it promotes EC adhesion and proliferation, prevents platelet and macrophage adhesion, and inhibits SMC proliferation [109]. Wang and colleagues [110] developed LBL coatings using oxidized HA (OHA) nanogels and PEI. The OHA layer was incorporated with rivaroxaban, an FXa inhibitor, and further crosslinked with a thrombin-responsive peptide, while the PEI layer was co-loaded with tempol and EGCG. Upon thrombin stimulation, rivaroxaban can be released from the nanogels, thereby increasing the hemocompatibility. On the other hand, tempol can scavenge free radicals and EGCG can further complement free radical inhibition via its anti-oxidative ability. Once used as coatings for bioprosthetic heart valves, this assembly improved anticoagulation and anti-calcification, as well as enhanced host integration and endothelium production at day 30 post-implantation. Moreover, these coatings improved reendothelialization, resolved inflammation, and suppressed neointimal growth in implanted stents at 1- and 3-month post-implantation in a rabbit abdominal aorta model.

### Cell membrane-based modifications

**Fig. 6.**
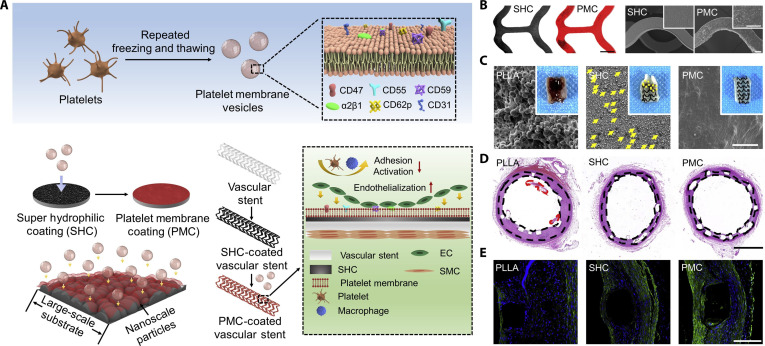
Anticoagulant and endothelium-promoting vascular stent modification based on platelet membrane coating (PMC). (A) Schematic of the PMC coating on a vascular stent. (B) Confocal laser scanning microscope (CLSM) and SEM images comparing the surface of SHC and PMC-coated vascular stents. (C) Morphology of in-stent thrombus 8 h post-implantation. Yellow arrows indicate the adhered platelets. (D and E) Histological analysis 30 d after implantation: (D) H&E staining and (E) IF staining for α-SMA (green) [111]. Copyright 2022, Elsevier.

Modification of biomaterials with cell membranes derived from diverse sources can confer multiple functionalities, including immune evasion, enhanced biocompatibility, and targeted specificity. These cell-derived membranes carry various functional proteins and biomolecules that facilitate tissue-specific targeting, immune evasion, and precise antigen presentation [82]. Zhou et al. [111] developed a platelet membrane coating (PMC) by fusing platelet membrane vesicles with a PDA-based and nanoparticle-stacked super-hydrophilic coating (SHC) PLLA sheet (Fig. 6A and B). This PMC retained phospholipids and preserved functional proteins, including CD47, CD55, CD59, and α2β1, and provided extended biological functionality compared to synthetic membrane mimics. In vitro, the coating reduced platelet adhesion and promoted EC growth while inhibiting SMC proliferation. In an ex vivo rabbit thrombogenicity test, PMC coating reduced occlusion and thrombotic deposition, and in a rat subcutaneous implantation model, it reduced inflammatory response and fibrous capsule formation. Moreover, in a rabbit abdominal aorta model, PMC-coated stents exhibited reduced thrombosis, resolved inflammation, and enhanced reendothelialization at 30 d post-implantation (Fig. 6C to E). Similarly, red blood cell membrane (Rm) was used to modify the surface of PCL- and poly-d-lysine-based SDVGs through co-incubation and one-step rolling of the SDVGs with Rm. The Rm exhibits anti-inflammatory and thromboprotective functions due to the presence of CD47, which can bind to signal regulatory protein α and lead to a reduction in inflammatory cell and platelet adhesion. The presence of poly-d-lysine can improve cell adhesion and enhance the interaction with Rm, which facilitates the stability of Rm on SDVGs. The modified SDVGs reduced fibrinogen adsorption and platelet adhesion, as well as decreased macrophage adhesion and tumor necrosis factor-α (TNF-α) levels, but increased IL-10 levels in vitro. Moreover, when used as carotid artery substitutes in rabbits, the modified grafts conferred immunomodulatory effects and induced EC and contractile SMC regeneration, and ECM remodeling, alongside improved patency at 21 d post-implantation [112].

In summary, various antithrombotic strategies, including heparin polysaccharides, polyzwitterions, ECM proteins, and cell membrane components, have been developed to enhance the thromboresistance and biocompatibility of vascular scaffolds (Table 2). Among these, heparin-based modifications and polyzwitterions demonstrate reliable anticoagulant performance and are relatively cost-effective, making them clinically favorable and widely adopted. However, for long-term patency and functional integration, scaffolds still require endothelium-inducing modifications, such as those incorporating ECM proteins or bioactive cell membrane derivatives, to promote endothelialization and improve overall therapeutic outcomes. Future efforts should focus on combining economical, antithrombotic modifications with targeted endothelial-promoting components to achieve both immediate thromboresistance and sustained vascular healing.

**Table 2.** Comparison of different antithrombotic modification strategies for vascular scaffolds and stents

Type of modification	Composition	Implant duration	Animal model	Results	Ref.
Polysaccharide	Heparin grafted on electrospun PEUU–gelatin graft	1 and 4 weeks	Rat abdominal aorta model	Prevented platelet adhesion and promoted EC adhesion/proliferation in vitro, enhanced ECs and SMCs regeneration in vivo	[80]
	Chitosan and heparin layers with embedded EGCG/Cu^2+^ complex	3 months	Rabbit abdominal aorta stent implantation model	Inhibited thrombosis, suppressed SMC proliferation and inflammation, and induced EC coverage and vascular healing	[81]
	Fucoidan immobilized in PVA hydrogel	2 and 4 weeks	Rabbit carotid artery model	Reduced platelet adhesion in vitro, enhanced in situ endothelialization to 60% compared to 0% in control group, and improved patency rate	[85]
Polyzwitterion	Copolymer spray-coated PLLA stents containing MPC, MDO, and BMA	42 d	Pig coronary artery model	Improved hemocompatibility, down-regulated IL-6 and MCP-1, prevented IH and collagen production	[90]
	pSB matrix reinforced with pCB microgels	Rabbit model—2 h; rat model—1 h	Rabbit AV shunt model; rat ECC model	Displayed strong adhesion, reduced occlusion, thrombus weight and protein adsorption, mitigated foreign body response	[96]
	PCBDA copolymer immobilized on a PDA–PEI pre-coated surface	1 and 3 months	Rabbit abdominal aorta stent implantation model	Reduced platelet and fibrinogen adhesion, reduced inflammation and IH, promoted EC growth while inhibited SMC proliferation	[100]
ECM components	rhCol III immobilized on PLA stents	3 months	Rabbit abdominal aorta and pig coronary artery model	Reduced ISR and neointimal area, enhanced endothelialization and supported M2 macrophage polarization	[107]
	Oxidized HA nanogel layer loaded with rivaroxaban; PEI layer incorporated with tempol and EGCG	1 and 3 months	Rabbit heart valve and abdominal aorta stent implantation model	In heart valves coating provided anticoagulation, anti-calcification, host integration, and endothelialization; in stents coating enhanced endothelialization, reduced inflammation and neointimal growth	[110]
Cell membrane components	Platelet membrane vesicle fusion onto PLLA sheets	1 month	Rabbit abdominal aorta stent implantation model	Reduced platelet adhesion, enhanced EC proliferation, inhibited SMC growth, and thrombotic occlusion in vitro*,* improved re-endothelialization, reduced thrombosis and inflammation in vivo	[111]
	RBC membrane adsorbed on PCL/poly-d-lysine- vascular graft	21 d	Rabbit carotid artery model	Reduced fibrinogen adsorption, platelet/macrophage adhesion, decreased TNF-α and increased IL-10 in vitro; enhanced EC and contractile SMC regeneration, ECM remodeling, and patency	[112]

PVA, polyvinyl alcohol; ECM, extracellular matrix; rhCol III, recombinant human type III collagen; PLA, polylactic acid; ISR, in-stent restenosis; EGCG, epigallocatechin gallate; PEI, polyethyleneimine; PDA, polydopamine; Cu^2+^, copper ions; SMC, smooth muscle cell; EC, endothelial cell; PLLA, poly(-l-lactic acid; PCBDA, poly(carboxybetaine acrylate–co–dopamine methacrylate); RBC, red blood cell; PCL, polycaprolactone; TNF-α, tumor necrosis factor-α; IL-10, interleukin-10; MCP-1, monocyte chemoattractant protein-1; MPC, 2-methacryloyloxyethyl phosphorylcholine; MDO, 2-methylene-1,3-dioxepane; BMA, butyl methacrylate; IH, intimal hyperplasia; ECC, extracorporeal circuit; AV, arteriovenous; pSB, poly(sulfobetaine)

## Endothelium-Inducing Modifications for Vascular Scaffolds

One leading cause of vascular graft failure is insufficient endothelialization [113]. This deficiency contributes directly to graft dysfunction, as inadequate EC regeneration can provoke late-stage thrombosis and initiate IH [114,115]. In native blood vessels, the endothelium can prevent thrombosis through physical and biochemical mechanisms. Its antithrombotic function is largely attributed to the glycocalyx—a hydrated, negatively-charged surface layer that repels platelets, leukocytes, and plasma proteins, while absorbing anticoagulant and antiplatelet molecules. Key components in the glycocalyx layer include HA, which forms a passive antifouling barrier via a robust hydration layer, and heparan sulfate, which actively inhibits coagulation factors (e.g., FXa and thrombin) by binding to antithrombin III. Transmembrane glycoproteins such as thrombomodulin and the endothelial protein C receptor further neutralize thrombin and enhance protein C activation, thereby reducing thrombin generation. Additionally, the endothelium secretes tissue-type plasminogen activator to degrade fibrin, tissue factor pathway inhibitor to limit thrombin, and NO and prostacyclin to inhibit platelet activation via calcium regulation [116–118].

Currently, several strategies have been employed to enhance endothelialization in implanted vascular scaffolds and grafts. One approach involves pre-implantation seeding of vascular scaffolds with autologous ECs, which avoids the need for drug-induced immunosuppression. However, its clinical adoption is limited by practical hurdles such as difficult cell harvest, limited proliferative capacity, time-consuming in vitro cell expansion, and vulnerability of EC layer to shear-induced detachment under physiological flow. As an alternative, in situ endothelialization strategies aim to foster the formation of a functional endothelium directly on the surface of vascular scaffolds, primarily through the following mechanisms: (a) transanastomotic migration of host ECs from the adjacent native vessels, (b) transmural EC ingrowth through new microvessels within the graft walls, and (c) fallout endothelialization, wherein circulating EPCs home to and colonize the graft [4]. To accelerate endothelial regeneration in vascular scaffolds, various surface modification strategies have therefore been explored, such as using bioactive peptides, cell adhesion or matrix proteins, growth factors/antibodies, and NO-releasing compounds.

### Bioactive peptide-based modifications

Since the surface density of an adhesive peptide sequence and the spacing between peptides can directly affect cell–ligand interactions, modification of vascular scaffolds with bioactive cues exhibiting anability to spatiotemporally present cell-adhesive cues holds great promise [119]. Indeed, RGD-functionalized copolymer-based modifications have been demonstrated to develop both random and nanoclustered surfaces with varying RGD densities. The density range of cell-adhesive RGD sequence (0.2 to 1.9 μg) per polymer was considered as a global density, while 1 to 2.4 ligands per cluster were considered as the local peptide density. HUVECs cultured on these peptide-functionalized surfaces displayed enhanced adhesion, migration speed, and proliferation, compared to the random surfaces, due in part to the maximum global and local peptide densities [120].

Zheng et al. [121] designed a triblock functional protein coating using a mussel-inspired adhesive peptide (YKYKY)_5_ that supported adhesion to the PTFE surface and a zwitterionic polypeptide (KE)_20_ for antifouling ability. The coating was further incorporated with EC-specific bioactive peptides (REDV and YIGSR) to promote EC adhesion while reducing the attachment and growth of SMCs. Since EC adhesion and growth are indispensable for endothelium health, and SMC overgrowth inhibition is important to suppress IH, these intelligent multifunctional protein-based modifications are worthy of future investigations. Moreover, Anderson and coworkers [122] covalently modified PVA films and vascular grafts with a collagen-mimicking peptide (GFPGER) to enhance EC adhesion while reducing thrombosis. The GFPGER peptide possesses an amino acid sequence similar to the GFPGOR motif found in the collagen, and both of these sequences can recognize α1β1 and α2β1 integrins, leading to the binding of ECs. The peptide-modified PVA scaffolds improved the confluence and vascular endothelial cadherin (VE-Cad) density of ECs, reduced platelet adhesion and activation, and delayed fibrin deposition in vitro*.* The efficacy of this approach requires further investigation via in vivo implantation of peptide-functionalized vascular grafts in animal models.

Since EPCs exhibit greater expansion and differentiation capacity than that of mature ECs, in situ mobilization and recruitment of EPCs using functional peptides has emerged as a promising strategy for inducing endothelium formation [123,124]. For example, Hao et al. [125] immobilized the LXW7 peptide (cGRGDdvc) on PCL/PLLA-based vascular grafts using click chemistry. The peptide-functionalized grafts appreciably reduced platelet adhesion but enhanced the proliferation, migration, and endothelial differentiation of endothelial colony-forming cells in vitro. More importantly, LXW7-modified grafts inhibited thrombus formation and promoted blood vessel regeneration and endothelialization by selectively capturing circulating EPCs. Compared with the control group, which showed a patency rate of only 17%, LXW7-conjugated grafts achieved patency rates of up to 83%, along with improved cell infiltration and vascularization. Structurally, LXW7 is an octapeptide composed of unnatural amino acids, which confers resistance to proteolytic degradation, a common limitation of linear peptides. It functions as a high-affinity ligand for α_v_β_3_ receptors on EPCs and ECs, enabling effective and specific cell attachment [126]. Unlike conventional RGD peptides (Arg–Gly–Asp), which nonspecifically bind not only to EPCs/ECs but also to inflammatory cells and platelets, the LXW7 peptide demonstrates selective recruitment of EPCs with minimal or undetectable binding to platelets and inflammatory cells.

### Cell adhesion and matrix protein-based modifications

Cell adhesion molecules play essential roles in mediating cell–cell and cell–ECM interactions. Major classes of these molecules include integrins, cadherins, and selectins [127]. In native blood vessels, EC adhesion is primarily mediated by VE-Cad, a transmembrane protein that forms adherens junctions between ECs. VE-Cad forms Ca^2+^-dependent homodimers between adjacent ECs and plays a fundamental role in regulating cell proliferation and endothelial barrier function. This barrier restricts the passage of cells and molecules across the vascular wall, thereby maintaining vascular permeability and inhibiting leukocyte extravasation and cell migration [128,129]. In a notable application, Lee et al. [130] modified PCL grafts with a truncated form of VE-Cad containing only the first 4 extracellular domains (EC1 to EC4) using PIII. These domains are known to be critical for regulating EC adhesion and intercellular interactions. In a mouse carotid artery model, the VE-Cad-modified grafts showed markedly higher endothelial coverage (~90% EC coverage) compared with control grafts 14 d post-implantation. Moreover, the modified grafts exhibited reduced fibrin deposition and attenuated macrophage accumulation.

VEGF and VE-Cad can synergistically regulate the organization of ECs during vascular sprouting, promote angiogenesis, and maintain vascular stability. To enhance EC adhesion, migration, and differentiation, Wang and colleagues [131] immobilized hVE-Cad-Fc and hVEGF-Fc fusion proteins onto PCL fibers. This dual-functionalization of vascular scaffolds enhanced VE-Cad expression, which positively regulated the behavior of ECs. Furthermore, these vascular grafts enhanced EC coverage and SMC regeneration in a rat abdominal model at 28 d post-implantation. Collectively, cell adhesion protein VE-Cad-based modifications capable of promoting EC survival, proliferation, and migration, as well as EPC recruitment and differentiation, possess significant potential to improve in situ blood vessel regeneration in vascular scaffolds.

Matrix proteins can promote endothelialization by providing both adhesive and signaling cues to ECs. Vascular stents and scaffolds modified with rhCol III have been shown to inhibit platelet activation and thrombosis while simultaneously enhancing endothelialization in vivo [106–108]. Although EPCs hold promise for reendothelializing vascular scaffolds, their scarcity in peripheral blood often limits in vivo endothelialization efficiency [132]. In contrast, late outgrowth endothelial cells (EOCs), a subtype of EPCs derived from peripheral blood, express characteristic endothelial markers and exhibit strong regenerative potential. To enhance EOC-specific adhesion, a research group engineered a streptococcal collagen-mimetic protein (Scl2) by incorporating a single α1β1 integrin-binding sequence (GFPGER), generating a designer collagen variant termed Scl2-GFPGER (also referred to as DC2-1X) [133]. Subsequently, the same team developed the derivatives containing multiple integrin-binding sequences, such as DC2-3X1, which markedly improved EOC retention under physiological shear stress [134]. Collectively, these findings highlight the potential of collagen-mimic proteins as promising endothelium-inducing materials for vascular scaffolds and stents, warranting further investigation.

### Growth factor/antibody-based modifications

Various types of growth factors, including VEGF, stromal cell-derived factor-1α (SDF-1α), basic fibroblast growth factor (bFGF), and platelet-derived growth factor (PDGF), have been employed to enhance in situ vascular regeneration by promoting the mobilization and recruitment of endogenous stem/progenitor cells (SPCs) and their differentiation into vascular cells [135]. Among these, VEGF is widely recognized as a key regulator of angiogenesis [136]. Importantly, vascular grafts loaded with VEGF and heparin can achieve rapid endothelialization and antithrombotic outcomes [137]. Previously, acellular tissue-engineered vessels (A-TEVs) based on small intestinal submucosa (SIS) functionalized with heparin and VEGF were fabricated and implanted into a mouse arterial model. VEGF-functionalized grafts achieved complete endothelialization within 1 month of implantation as well as displayed superior remodeling, reduced inflammation, and maintained patency. Mechanistically, VEGF promoted the conversion of infiltrating monocytes into anti-inflammatory M2 macrophages and induced cells with dual macrophage–endothelial phenotypes [138]. The same group also implanted A-TEVs into an ovine arterial model, where the A-TEVs achieved complete endothelial coverage and remained patent for up to 1 and 3 months. The immobilized VEGF selectively captured monocytes under physiological shear stress and induced their differentiation into a hybrid macrophage–endothelial phenotype that matured into NO-producing ECs aligned with blood flow [139].

Wang et al. [140] developed a bilayer vascular scaffold to obtain controlled release of growth factors to promote vascular regeneration. The inner layer of the scaffold, composed of electrospun PLCL/collagen nanofibers loaded with heparin and VEGF, supported rapid endothelium formation, while the outer layer composed of circumferentially aligned PLCL/collagen nanofiber yarns incorporated a gradient distribution of PDGF, which encouraged SMC alignment and infiltration. Following implantation in a rat abdominal aorta model, the scaffold displayed complete endothelialization, organized SMC layers, and collagen-rich connective tissue deposition within 2 months. Fahad et al. [141] fabricated core–shell fibrous SDVGs via co-axial electrospinning, where the shell, composed of PCL/gelatin loaded with heparin and VEGF, facilitated rapid degradation and sustained release of heparin and VEGF, while the PCL core maintained long-term structural integrity of vascular grafts. These SDVGs demonstrated superior biocompatibility and hemocompatibility, and in vivo implantation in a rat abdominal aorta model showed complete endothelialization, SMC regeneration, and 100% patency after 4 months without developing IH.

**Fig. 7.**
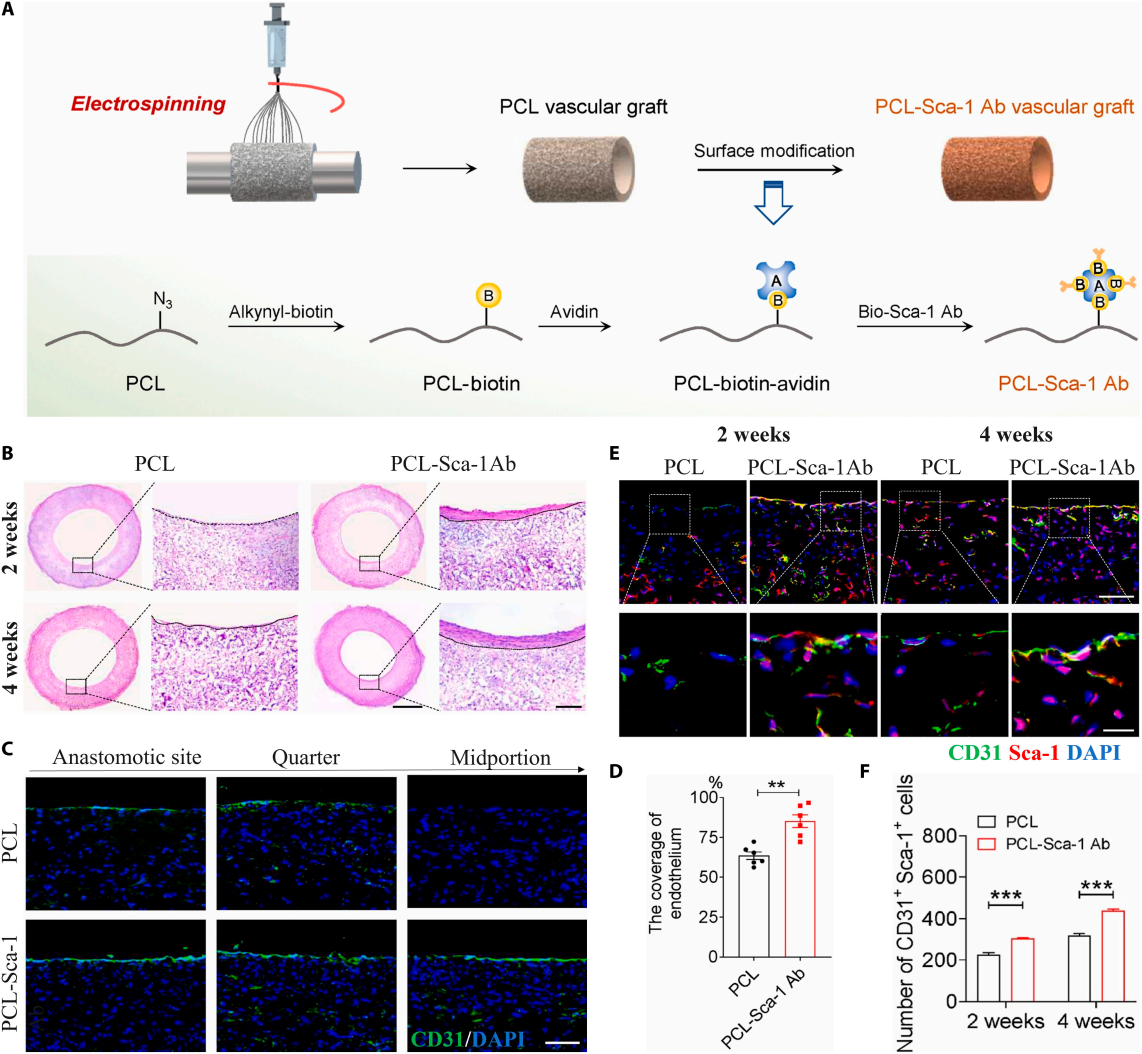
Design and vascular regenerative effects of anti-Sca-1 antibody-modified vascular grafts. (A) Schematic shows the procedure for immobilization of anti-Sca-1 antibodies on electrospun PCL vascular grafts. (B) H&E staining images indicate tissue regeneration within the vascular grafts. Neo-tissue is bordered by dashed lines. Scale bars, 1 mm or 100 μm (magnified images). (C) CD31 IF staining images of longitudinal sections of the implanted grafts, and (D) quantitative analysis of endothelium coverage. Scale bar, 100 μm. (E) Co-IF staining of Sca-1 and CD31 in the vascular graft wall at 2 and 4 weeks post-implantation. Scale bars, 100 μm or 20 μm (magnified images). (F) Quantification of Sca-1^+^ CD31^+^ cells in the vascular grafts [142]. Copyright 2022, KeAi.

Despite the advantages of functionalizing vascular grafts with growth factors, their susceptibility to degradation in proteolytic tissue microenvironments, along with the challenges of synthesis, purification, and incorporation into scaffolds, limits their widespread application. Alternatively, protein-based candidates such as antibodies offer greater stability and they can be more readily produced and integrated into vascular grafts. Antibodies can recognize specific antigen-presenting cells, allowing the development of biomaterials that capture and recruit target cells, thereby enhancing scaffold cellularization and promoting tissue regeneration. Wang et al. [142] developed an electrospun PCL vascular graft functionalized with stem cell antigen-1 (Sca-1) antibodies using a biotin–avidin immobilization strategy to promote endogenous SPC recruitment (Fig. 7A). The modified grafts effectively captured and retained Sca-1^+^ SPCs under static and dynamic flow conditions in vitro. Moreover, in the rat abdominal aorta model, the modified grafts developed well-organized neo-tissue formation (Fig. 7B) as well as they induced rapid endothelialization (Fig. 7C and D) and SMC regeneration. Importantly, the source of infiltrated and regenerated ECs was Sca-1^+^ SPCs, which were partially differentiated into ECs (Fig. 7E and F). Similarly, an ePTFE vascular graft was functionalized with a heparin/collagen multilayer incorporating anti-CD133 antibodies to accelerate early endothelialization. The modified grafts not only revealed enhanced hemocompatibility and endothelial-promoting abilities but they also promoted rapid endothelial coverage of the lumen surface in a porcine carotid artery model [143].

Since EPCs can enhance rapid endothelialization and long-term patency rates in synthetic vascular grafts, a CD133^+^ EPC capture surface was engineered by co-immobilizing anti-CD133 antibody, REDV peptide, and VEGF onto a hybrid methacrylated HA (MA-HA)/heparin antifouling layer. The functionalized surface presented dual functionality of heparin for VEGF conjugation and MA-HA for bioactive molecule anchoring. This surface showed excellent hemocompatibility and selectively captured CD133^+^ EPCs from peripheral blood mononuclear cells, as well as enhanced EPC proliferation and endothelial differentiation through integrin–VEGF receptor (VEGFR) synergistic signaling [123]. Sun et al. [144] developed a functionally segmented SDVG using emulsion electrospinning of thermoplastic PU and collagen, incorporating multiple bioactive molecules to simultaneously address anticoagulation, endothelialization, and SMC proliferation. The entire graft was loaded with heparin and anti-CD133 antibody to promote endothelial growth and reduce thrombosis, while rapamycin was incorporated at both ends to inhibit SMC overprolifertion and prevent IH. These modified grafts revealed rapid endothelialization, effective anticoagulation, and inhibition of SMC proliferation in rabbit carotid arteries.

### NO-based modifications and others

NO plays a crucial role in maintaining vascular homeostasis and tissue repair through its pro-angiogenic and vasculogenic effects. Under physiological conditions in native blood vessels, NO produced by endothelial NO synthase (eNOS) preserves an anti-inflammatory and anticoagulant microenvironment by inhibiting platelet activation, SMC proliferation, inflammation, thrombus formation, and ISR. Upon vascular injury, NO release enhances vascular regeneration by promoting EC repair and limiting IH [145–147].

Due to the short half-life of NO as a gaseous molecule, the functionalization of biomaterials with NO donors, such as RSNOs, has been leveraged for controlled and sustained release of NO in biomedical devices [148]. The promising RSNOs for preclinical studies include S-nitroso-N-acetylpenicillamine (SNAP) and S-nitrosoglutathione (GSNO). Exogenous or endogenous RSNOs can be catalytically decomposed to produce NO. Indeed, different types of catalytic agents have been explored to induce the degradation of RSNOs, including transition metal ions (e.g., copper and selenium) [149,150]. Importantly, these processes mimic the NO release function of ECs that naturally produce NO through eNOS for multiple protective functions, such as the regulation of vascular tone, inhibition of SMC overproliferation, and thromboprotection [151]. Therefore, modifications capable of sustainably releasing NO in situ may help promote endothelial function for NO-mediated vascular homeostasis.

**Fig. 8.**
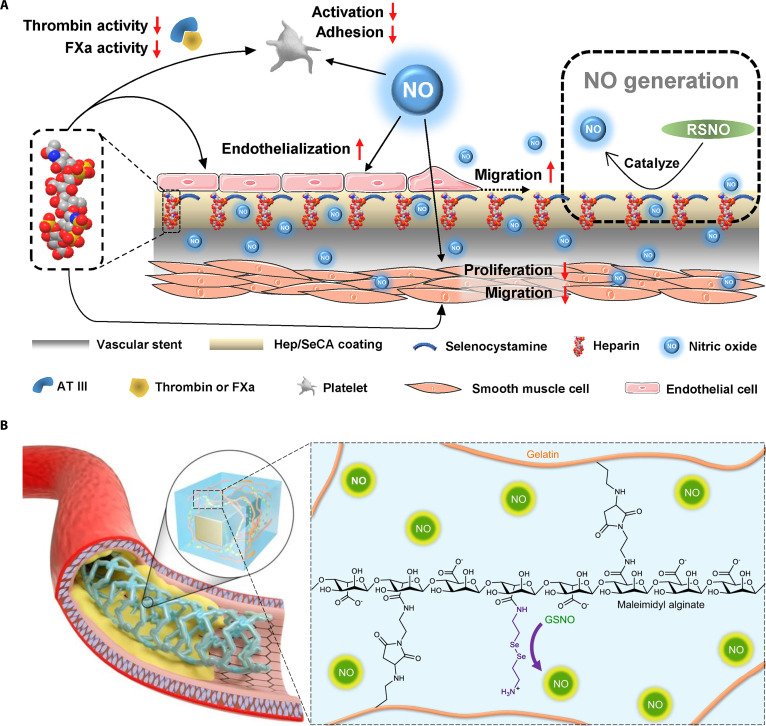
Nitric oxide-based modifications for the regulation of endothelial repair and hemocompatibility of vascular devices. (A) Schematic illustrates the development of an endothelium-like modification through dual application of bioactive heparin and NO-generating selenocystamine [152]. Copyright 2029, Elsevier. (B) Schematic shows the formation of NO-eluting hybrid hydrogel by the cross-linking between gelatin and maleimide-modified alginate [156]. Copyright 2021, Springer Nature.

In a previous study, a sequential co-immobilization strategy was used to create an endothelium-mimicking surface by covalently conjugating heparin and selenocystamine (SeCA) onto a plasma polymerized allylamine (PPAam)-coated stent [152]. The modification conferred thromboprotection, enhanced endothelium regeneration, and inhibited the proliferation of SMCs, because of the synergistic effect of SeCA and heparin. The SeCA exhibits glutathione peroxidase (GPx)-like activity; selenium-containing groups of SeCA can catalytically decompose endogenous RSNOs, leading to the controlled and sustained release of NO from the stent surface. On the other hand, as a mimic of the heparan sulfate, heparin can inhibit the coagulation cascade and suppress SMC proliferation. Collectively, heparin and SeCA provide a synergistic effect: Heparin provides anticoagulant properties, while SeCA catalyzes NO release, which inhibits platelet aggregation and further enhances antithrombotic and endothelial-protective effects. This synergy recapitulates native endothelial function and prevents ISR and late stent thrombosis. Furthermore, this modification strategy also up-regulated α-smooth muscle actin (α-SMA) expression and increased cyclic guanosine monophosphate (cGMP) synthesis, which facilitated the contractile phenotype of SMCs while preventing their proliferation (Fig. 8A). There are 2 main phenotypes of VSMCs: contractile and synthetic. Contractile VSMCs are found in the tunica media, where they regulate vascular tone and maintain blood vessel diameter by contracting in response to physiological signals. On the other hand, synthetic VSMCs are mainly located in the tunica intima and adventitia, and are involved in vascular repair and remodeling by increasing protein synthesis and proliferating in response to vascular injury [153]. α-SMA is a key component of the cytoskeletal filaments that regulates the contractile function of SMCs. The knockdown of α-SMA can disrupt the differentiation of SMCs, which promotes a dedifferentiated or synthetic phenotype of SMCs and leads to various vascular pathologies, including restenosis, hypertension, and atherosclerosis [154,155]. In a similar study, SeCA-conjugated gelatin and alginate-maleimide-based hydrogels released NO in situ by endogenous RSNO and conferred antithrombotic effects in an ex vivo AV shunt model, as well as prevented IH and enhanced endothelialization in rabbit iliac artery and porcine coronary artery models at 3 months post-implantation (Fig. 8B) [156]. These beneficial effects were due to the regulation of the cGMP–protein kinase G (cGMP-PKG) signaling pathway, which plays a crucial role in vascular biology. This pathway is involved in various cellular processes, including the regulation of eNOS, actin reorientation, focal adhesion, and migration of vascular cells. In addition, the cGMP-PKG pathway can also interact with other signaling cascades, such as protein kinase A, extracellular signal-regulated kinase, and ras-related protein 1, thereby regulating platelet aggregation, VSMC contraction, and EC proliferation [157–159]. In a related approach, a polyphenol–polyamine matrix composed of tannic acid and polyamines has been functionalized with SeCA and VEGF and shown to effectively promote vascular regeneration and immunomodulation in a rat abdominal aorta model for up to 1 and 3 months [160].

The exogenous supplementation of NO donors is a promising approach to functionalize the lumen of vascular grafts and stents. SNAP has also been used for localized and controlled release of NO in 3D-printed PLA vascular grafts. These PLA-based SDVGs were coated with a polyethylene glycol (PEG)/PCL matrix containing SNAP for sustained NO release. The modified grafts showed NO release for up to 14 d, enhanced EC proliferation and migration, inhibited bacterial growth, and augmented angiogenesis in vivo in a chorioallantoic membrane model [161]. This approach exhibits the potential to regulate EC function alongside antibacterial activity through sustained NO release from biomaterial platforms.

**Fig. 9.**
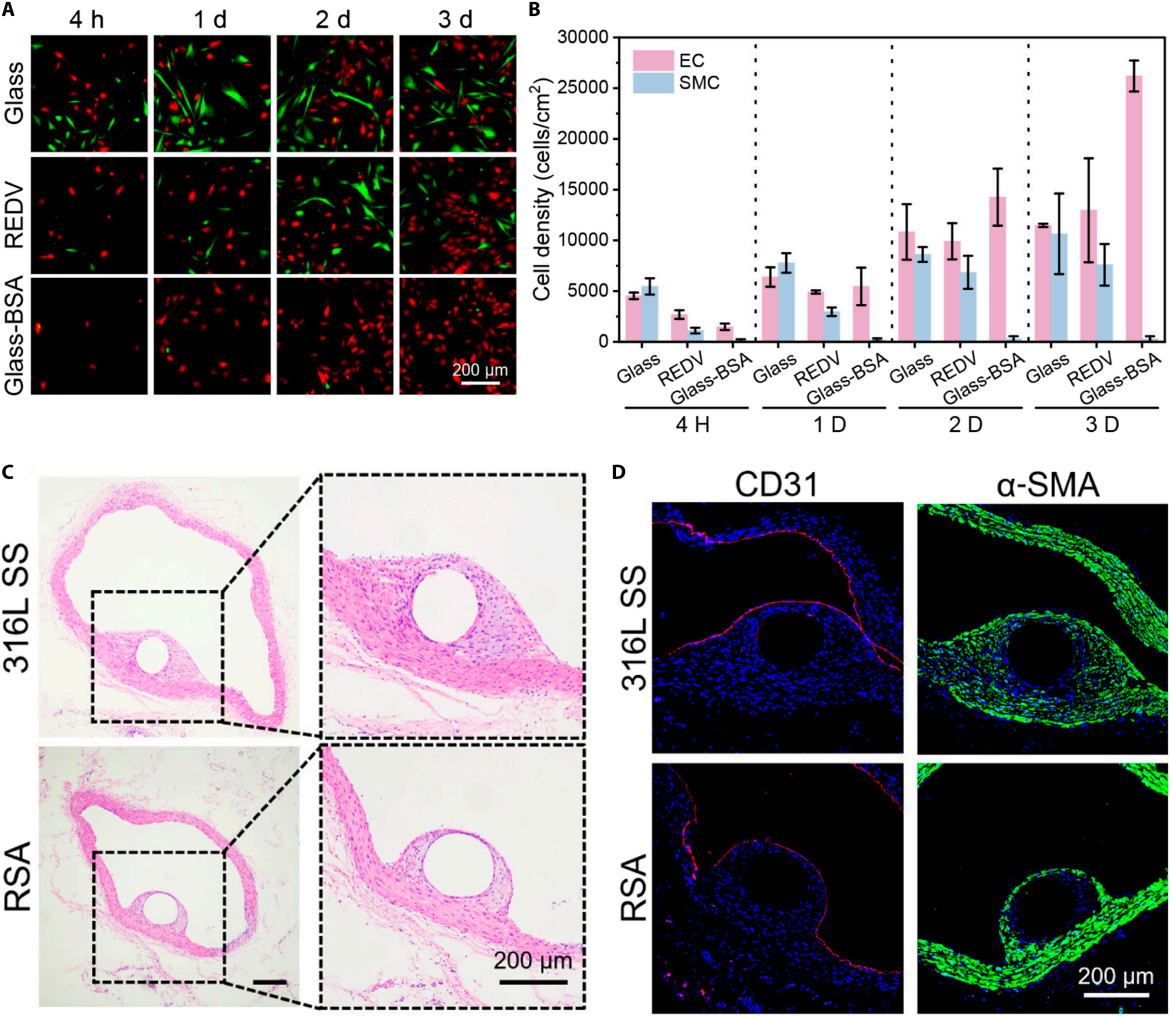
Albumin surface modification enhancing EC-selective adhesion. (A) Fluorescence images and (B) corresponding cell densities of ECs (red) and SMCs (green) adhered and proliferated on bovine serum albumin (BSA)-coated glass substrates. (C) Representative H&E staining images and (D) IF staining images indicate CD31 (red), α-SMA (green), and nuclei (blue) at 30 d post-implantation of rat serum albumin (RSA)-coated 316L stainless steel (SS) implants in a rat abdominal aorta model [162]. Copyright 2025, American Chemical Society.

Particularly, achieving selective EC adhesion over SMC adhesion on vascular graft and stent surfaces is critical for preventing late-stage thrombosis and restenosis. Recently, a study reported that an albumin-modified glass substrate exhibited ultrahigh EC selectivity, with an EC/SMC ratio exceeding 200 in a medium containing 5% fetal bovine serum. This ratio is significantly higher than other existing endothelial-promoting strategies, such as peptides, matrix proteins, antibodies, and NO-releasing molecules, which typically yield EC/SMC ratios below 10. Moreover, in a rat abdominal aorta model, albumin-coated stents promoted endothelization while suppressing IH formation 30 d post-implantation (Fig. 9) [162]. Nevertheless, further studies are needed to assess the clinical potential of this approach for implantable cardiovascular devices.

Overall, various antithrombotic modification strategies, including polysaccharides, polyzwitterions, ECM components, and cell membrane derivatives, have been explored to mitigate acute platelet activation and thrombosis in vascular scaffolds (Table 2). In parallel, a range of endothelium-inducing strategies, including bioactive peptides, cell adhesion and matrix proteins, antibodies, growth factors, and NO-releasing molecules, have been employed to enhance EC adhesion and endothelialization (Table 3). Notably, integrating both antithrombotic and endothelium-promoting functionalities onto cardiovascular implants may simultaneously suppress thrombosis and accelerate the formation of a functional endothelium. This dual-functional strategy may offer long-lasting hemocompatibility, improved long-term patency, and overall performance of implanted vascular devices. Considerably, a dynamic, real-time monitoring of vascular scaffolds would be crucial for postoperative management. In the following section, we will review recent advances in the integration of bioelectronics in vascular scaffolds for real-time, noninvasive monitoring of blood flow, thrombosis, and restenosis.

**Table 3.** Comparison of different endothelium-inducing modification strategies for vascular scaffolds and stents

Type of coating	Components	Implant duration	Animal model	Results	Ref.
Bioactive peptides	LXW7 peptide functionalization via click chemistry	6 weeks	Rat carotid artery	Induced EPC/EC recruitment, enhanced endothelium regeneration, inhibited thrombotic deposition and increased patency rate to 83% vs. 17% in control group	[125]
Cell adhesion and matrix proteins	VE-Cad (EC1–EC4) functionalized PCL vascular grafts	14 d	Mouse carotid artery	Enhanced attachment and proliferation of EC in vitro, induced EC coverage to 90%, reduced fibrin deposition and macrophage accumulation in vivo	[130]
	VE-Cad-Fc and VEGF-Fc dual fusion proteins	28 d	Rat abdominal aorta	Reduced thrombosis and IH, promoted endothelialization and SMC regeneration	[131]
Growth factors/antibodies	Acellular SIS graft with heparin and VEGF	1 month	Mouse descending aorta model	Induced rapid endothelialization, promoted vascular remodeling, reduced inflammation, promoted M2 macrophages, and maintained patency	[138]
	Acellular SIS graft with heparin and VEGF	1 and 3 months	Ovine carotid artery model	Achieved complete endothelial coverage and maintained patency, captured monocytes under shear stress, and induced their differentiation into NO-producing ECs	[139]
	Electrospun PCL graft with Sca-1 antibodies	4 weeks	Rat abdominal aorta	Encouraged rapid endothelialization and SMC regeneration, increased recruitment of Sca-1^+^ stem/progenitor cells, enhanced M2 macrophages and ECM remodeling	[142]
	PU/collagen graft with heparin, anti-CD133, and rapamycin	1 and 2 months	Rabbit carotid artery model	Grafts achieved full patency with no thrombosis, promoted complete endothelialization, and suppressed SMC proliferation	[144]
NO-releasing molecules	Heparin conjugated with SeCA on allylamine film	1 and 3 months	Rabbit iliac artery	Reduced platelets and fibrinogen adhesion and activation, enhanced EC growth, while suppressed SMC proliferation	[152]
	Gelatin and maleimide alginate conjugated with SeCA	3 months	Rabbit iliac artery and porcine coronary artery	Provided anti-thrombotic effects ex vivo, inhibited IH, enhanced endothelialization and vascularization in vivo	[156]
	SNAP-loaded PLA-based grafts with PCL top-coat	14 d	CAM model	Sustained NO release; enhanced EC migration, antibacterial effect, and angiogenesis	[161]

LXW7 peptide, cGRGDdvc; EPC, endothelial progenitor cell; EC, endothelial cell; SIS, small intestinal submucosa; VEGF, vascular endothelial growth factor; NO, nitric oxide; SCA-1, stem cell antigen-1; ECM, extracellular matrix; SMC, smooth muscle cell; IH, intimal hyperplasia; VEGF-Fc, vascular endothelial growth factor fused to the Fc region of IgG_1_; VE-Cad-Fc, vascular endothelial-cadherin fused to the Fc region of IgG_1_; VE-Cad (EC1–EC4), vascular endothelial-cadherin extracellular domain 1 to 4; PU, polyurethane; SeCA, selenocystamine; NO, nitric oxide; SNAP, S-nitroso-N-acetyl-d-penicillamine; PLA, polylactic acid; CAM, chorioallantoic membrane

## Intelligent Monitoring of Vascular Scaffolds

Intelligent monitoring represents a transformative strategy to enhance the functionality and clinical surveillance of vascular scaffolds. The integration of flexible electronics with wireless energy harvesting and data transmission capabilities enables real-time tracking of key hemodynamic parameters such as pressure, flow, and strain [163–165]. These electronic components, when incorporated into tissue-engineered scaffolds, allow continuous sensing and precise feedback or stimulation, thereby influencing the cellular differentiation and proliferation, and promoting tissue repair and regeneration. Conformal and flexible sensing technologies can detect subtle changes in the arterial wall, delivering high-resolution data for cardiovascular monitoring and allowing early detection of pathological cell growth [166–168]. For example, smart stents embedded with wireless sensors can facilitate noninvasive, real-time monitoring of intravascular conditions, thus supporting early diagnosis and timely intervention [169]. Additionally, electronically integrated wearable devices, such as single-lead electrocardiogram sensors, offer further opportunities for early detection of acute cardiovascular events like coronary artery syndrome [170].

**Fig. 10.**
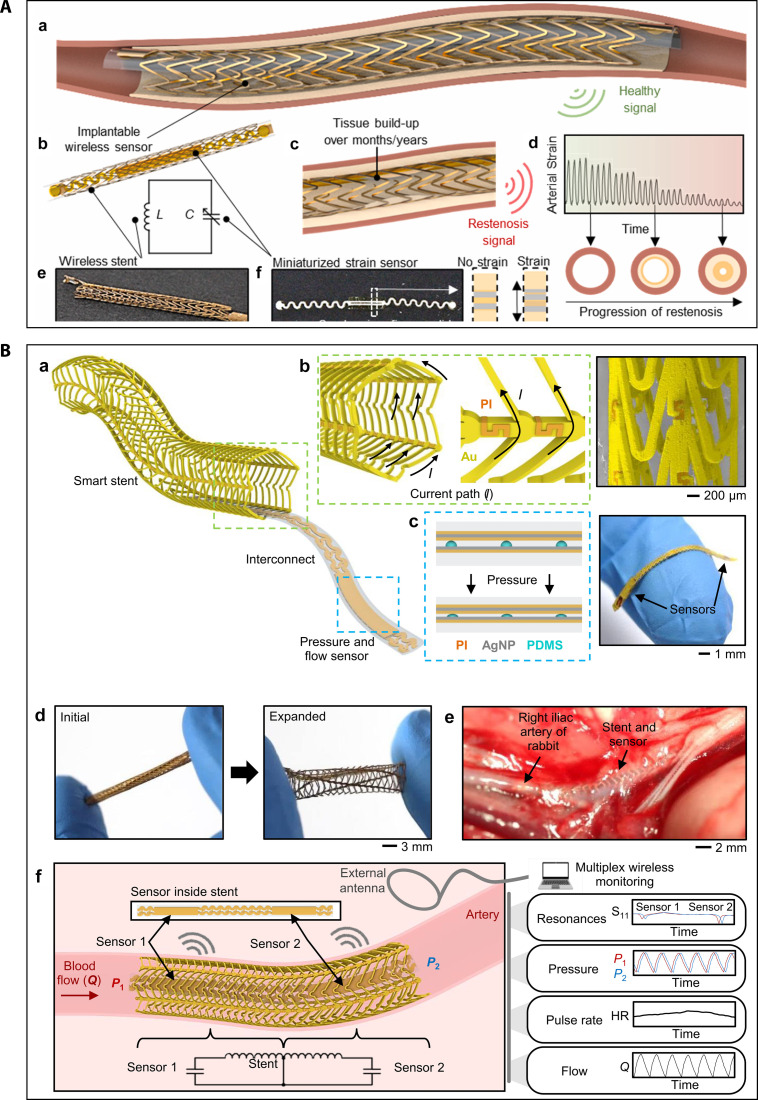
Design, fabrication, and functional evaluation of bioelectronic-integrated vascular devices for real-time hemodynamic and restenosis monitoring. (A) (a) Illustration of the wireless vascular device implanted in an artery. (b) Diagram of an implantable device consisting of a wireless stent and a capacitive strain sensor with a correlating LC circuit. (c) Depiction of restenosis occurring within the implanted stent. (d) Simulated wireless signals captured over time to identify the progression of restenosis. (e) Photograph of the wireless stent. (f) Miniaturized strain sensor indicating the effects of strain in finger alignment [172]. Copyright 2023, Elsevier. (B) (a) Illustration of the implantable electronic components. (b) Inductive stent design using conductive gold (Au) loops and non-conductive polyimide (PI) connectors to achieve a current path resembling a solenoid (left) and an SEM image of the stent (right). (b) Layers of the soft pressure sensor using a printed dielectric layer (left) and a photo of an index finger holding a simultaneous flow and pressure sensor (right). (d) Initial and expanded state of the sensor-integrated stent system. (e) A wireless stent system implanted in the right iliac artery of a living rabbit. (f) Illustration of the wireless design and sensing scheme to simultaneously monitor pressure, heart rate (HR), and flow [173]. Copyright 2022, American Association for the Advancement of Science.

Battery-free wireless vascular electronic systems have further expanded possibilities for continuous hemodynamic monitoring via Bluetooth low energy (BLE) communication [171]. For instance, Rigo et al. [172] developed a miniaturized printed bioelectronic system that combines a soft strain sensor with an inductive stent to support long-term, wireless detection of restenosis. The device operated through an LC circuit was formed by coupling the capacitive sensor with an inductive stent, facilitating prolonged monitoring of mechanical changes caused by the tissue overgrowth. By wirelessly capturing arterial strain signatures, the system achieved high-sensitivity tracking of restenosis progression (Fig. 10A). Similarly, another group developed an implantable, wireless, and batteryless vascular stent electronic system with printed soft pressure sensors for hemodynamic monitoring. The wireless stent was formed by conductive gold (Au) loops and nonconductive S-shaped polyimide (PI) connectors to achieve a conductive pathway resembling a solenoid and serve as an inductive antenna. The soft pressure sensor was printed with the layers of PI, silver nanoparticles (AgNPs), and dielectrical layer of polydimethylsiloxane (PDMS) lines. Thus, the integrated stent and soft capacitive sensors can form inductor–capacitor (LC) circuits with a resonant frequency dependent on pressure. The integrated sensor remained functional during balloon expansion at 14 atm for 30 s, with only a 3.5% capacitance change after 60 s. Wireless operation was achieved through inductive coupling with stable signal transmission over 5.5 cm in air and 3.5 cm in blood, maintaining both mechanical integrity and biocompatibility in simulated and in vivo rabbit iliac artery models (Fig. 10B) [173].

An angioplasty-compatible smart stent was developed for wireless, real-time detection of ISR and occlusion. It integrated a micro-electromechanical systems (MEMS) capacitive pressure sensor with a helical stainless steel antenna to form a passive LC circuit, converting local hemodynamic pressure changes into shifts in resonant frequency without onboard power. Laser microwelding, thick gold electroplating, and parylene C encapsulation enhanced mechanical robustness, electrical quality, and compatibility with standard percutaneous procedures. While bench tests showed reliable wireless pressure sensing with ~12.4 mmHg resolution and superior signal quality, in vivo studies in a swine bypass model confirmed detection of clot-induced occlusion and continuous monitoring across a clinically relevant range of >100 mmHg [174]. In a related study, a multifunctional bioresorbable electronic stent integrating transient electronics and therapeutic nanomaterials was developed to facilitate simultaneous endovascular monitoring and treatment. The stent combined a magnesium-based bioresorbable platform with flexible nanomembrane flow and temperature sensors, on-site bioresorbable memory, and wireless power and data transmission using the stent itself as an antenna. It also incorporated ceria nanoparticles to mitigate oxidative stress and gold nanorod–mesoporous silica nanoparticles for near-infrared-triggered, on-demand drug release. This stent demonstrated reliable physiological sensing under mechanical deformation, effective wireless communication and data storage, reduced inflammation, and controlled localized therapy [175]. Similarly, a fully implantable, batteryless, wireless bioelectronic stent system was developed for continuous monitoring of arterial stiffness to track restenosis. The stent design employed a flexible, aerosol jet-printed capacitive strain sensor of AgNPs and PI with a multimaterial inductive stent, forming an LC circuit whose resonant frequency reflected arterial wall strain. This low-profile, conformal stent-sensor platform supported balloon-catheter deployment and was validated in biomimetic coronary artery models and ex vivo ovine hearts, where it accurately detected restenosis progression (0% to 90%) via changes in capacitance and wireless resonance [176]. One study reported a nanomembrane sensor-integrated coronary stent that supported wireless, continuous monitoring of arterial wall strain to track ISR. The flexible, low-profile capacitive sensor, together with the stent acting as an inductive coil, formed a system capable of detecting subtle changes in arterial compliance under pulsatile flow. By combining finite element analysis, computational fluid dynamics, and in vitro silicone artery models, the study demonstrated that arterial strain decreased with restenosis progression and that these changes could be reliably measured and transmitted in real time [177].

Based on the design of mechanically compliant and multifunctional vascular sensors, a 3-layered piezoelectric vascular graft was fabricated by sandwiching silver nanowire-integrated polyvinylidene fluoride (PVDF) mats between electrospun PCL layers. This structure displayed mechanical properties similar to those of native arteries, with its porous PCL inner layer facilitating endothelialization and the middle PVDF layer enabling accurate detection of blood pressure and pulse waveforms in rabbit carotid artery models [178]. Tang et al. [179] reported an implantable vascular electronic system for long-term hemodynamic monitoring, which was composed of a circumferentially aligned PVDF piezoelectric sensor encapsulated by a bioinspired, elastic PU “growable” sheath. This adaptive sheath accommodated arterial expansion while maintaining sensor sensitivity, preventing constriction, and allowing simultaneous monitoring of multiple physiological parameters such as blood pressure, heart rate, and respiration. The wireless, battery-free configuration minimized infection risk and maintenance needs, making the vascular electronic system a promising option for postoperative management in CVD patients, which may also be potentially adaptable for other implantable bioelectronic applications. Park et al. [180] introduced a self-healing and anti-thrombotic vascular conduit based on a tough self-healing polymer (T-SHP) coated with a lubricious, anti-fouling layer and integrated with a T-SHP/carbon nanotube-based biosensor. This multifunctional design provided tunable mechanical strength, high protein adsorption resistance, and excellent fluid repellency. The conduit prevented leakage under dynamic flow in an ex vivo porcine aortic model and exhibited good integration when applied to a rat inferior vena cava. Furthermore, the embedded biosensor accurately detected blood flow rates between 10 and 100 ml/min with preserved electrical integrity after repeated punctures, demonstrating exceptional durability for long-term vascular monitoring.

**Fig. 11.**
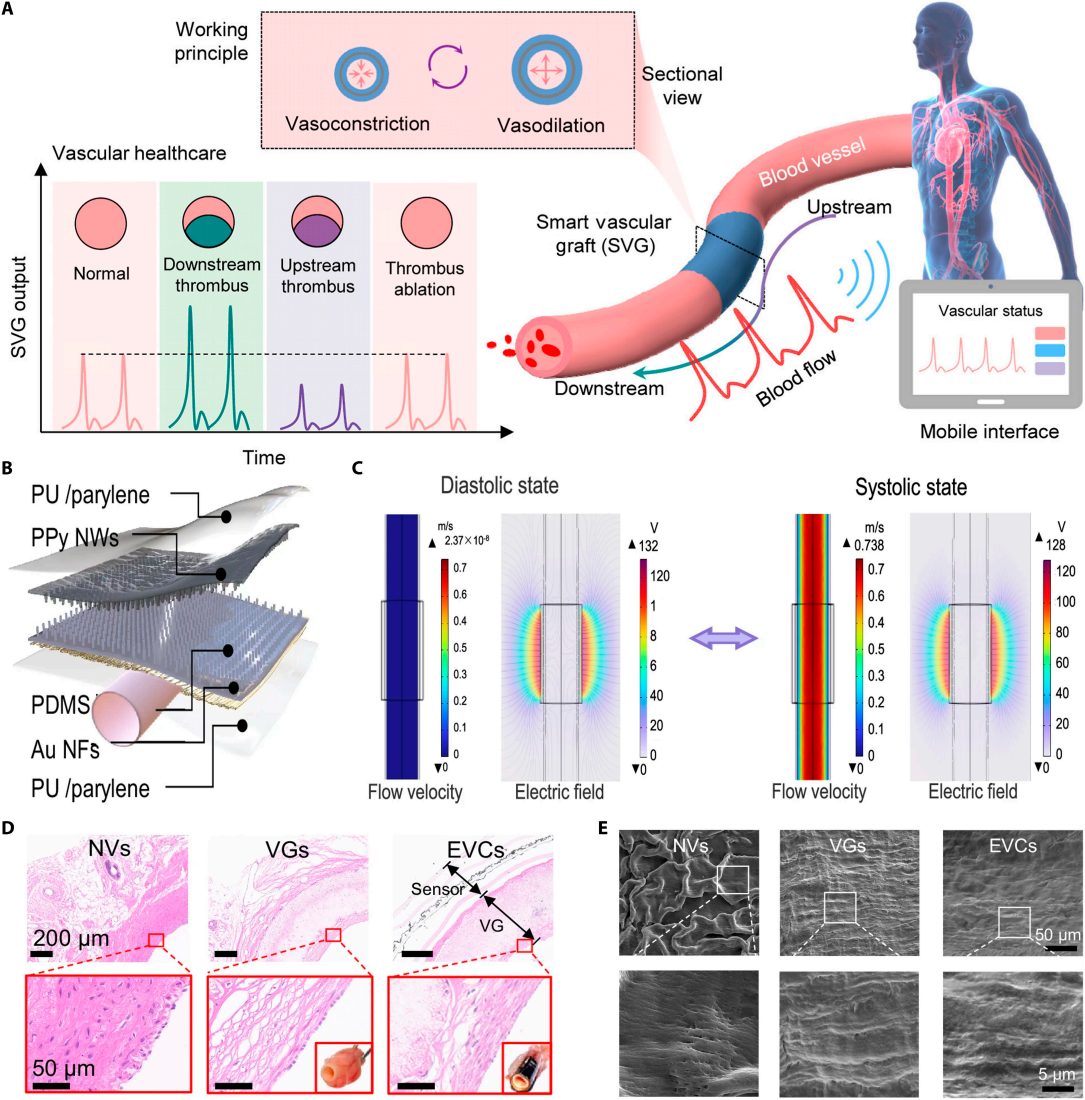
Design and working mechanism of smart bioelectronic-integrated vascular scaffolds for real-time hemodynamic sensing and in vivo remodeling. (A) Schematic illustration of the smart vascular graft, which expands during vasodilation and returns to its original size during vasoconstriction; the bottom left schematic indicates the output of the graft in response to different thrombi and their simulated therapy procedures [181]. Copyright 2025, American Chemical Society. (B) The diagram shows an electronic vascular conduit composed of polypyrrole and polydimethylsiloxane (PDMS) triboelectric layers with a backside gold nanofibrous electrode (AuNF), encapsulated in a PU/parylene film. (C) The sensor operates via triboelectric effect and electrostatic induction, where cyclic contact and separation of friction layers during systole and diastole generate periodic electrical signals. (D) H&E staining of cross-sections from different grafts at 3 months post-implantation. Inset: Optical images of the explanted grafts. (E) SEM images of the lumen surface of the grafts. NVs, native vessels; VGs, vascular graft; EVCs, electronic vascular conduit [182]. Copyright 2025, Springer Nature.

Currently, the integration of bioelectronics has expanded into monitoring key biological processes that determine the long-term function of the implanted vascular scaffolds. Recent studies have focused on intelligent vascular scaffolds capable of dynamically monitoring endothelial regeneration and restenosis. Such systems can sense both hemodynamic and cellular-level events, providing early diagnostic insights into pathological progression. Ma and colleagues [181] developed vascular grafts composed of porous laser-induced graphene between the top and bottom layers of PDMS. These grafts presented multifunctional characteristics and supported precise detection of blood flow changes, thrombus formation, and diagnosis of vascular blockages, thrombolytic drug response, and induced endothelialization. The system also allowed reliable remote monitoring through a wireless and portable version (Fig. 11A). In another study, a bionic vascular conduit integrated with flexible electronics was developed to facilitate long-term monitoring of blood flow, stenosis, and thrombosis. The system employed a polypyrrole (PPy)-based triboelectric generator with PDMS and nanofibrous gold electrode (AuNF) layers, all encapsulated in a PU/parylene C film to prevent biofluid interference (Fig. 11B). Based on triboelectric and electrostatic induction mechanisms, arterial pulsation generated measurable electrical signals (Fig. 11C). Real-time hemodynamic signals were transmitted wirelessly via BLE and visualized on a mobile interface, demonstrating reliable monitoring in both rabbits and cynomolgus monkeys. These grafts remained patent for up to 3 months in a rabbit carotid artery model and formed a continuous neotissue layer with a confluent endothelium on the lumen surface (Fig. 11D and E) [182]. In a related work, a smart triboelectric bilayer vascular graft was designed by fabricating a dual-layer scaffold using salt leaching and electrospinning. The inner layer consisted of a highly porous poly(glycerol sebacate) (PGS)/PCL composite loaded with the anti-thrombogenic drug “dipyridamole”. On the other hand, the outer sheath was an electrospun PCL/silk fibroin membrane incorporating the pro-endothelial drug “resveratrol”. This design ensured synchronized scaffold degradation with native vessel remodeling and sustained dual-drug release. The graft displayed superior mechanical properties, excellent hemocompatibility, and promoted biological functions, including increased EC migration, tube formation, and collagen production, alongside reduced SMC overproliferation. Particularly, the integrated triboelectric nanogenerator functionality supported real-time wireless monitoring of hemodynamic parameters, including flow and pressure, with a linear electrical output response [183]. In another study, sensor-integrated AV grafts (AVGs) were developed for electrical impedance spectroscopy as well as a radiotelemetry system for wireless signal transmission. These grafts were shown to successfully detect blood clots and emboli in swine carotid models alongside the identification of vascular cells in vitro. This strategy may help inform venous stenosis and blood clot formation in vivo, which could be applied in various types of implanted vascular devices [184].

The integration of electronics may also be closely related to the mechanical properties and biocompatibility of vascular scaffolds. The incorporation of flexible electronic materials, including graphene, MXene, silver nanowires (AgNWs), and silver (Ag) flakes, can support precise integration with vascular scaffolds without a distinct loss of the mechanical properties of vascular scaffolds. As discussed above, piezoelectric vascular grafts have demonstrated excellent mechanical characteristics, such as axial and radial strength, compliance, and burst pressure, even comparable to those of the native blood vessels. These scaffolds have also shown cytocompatibility and hemocompatibility, suggesting their potential implications for vascular monitoring [178]. Besides mechanical stability, the biocompatibility of electronic materials is also a major concern, especially for long-term implantations of vascular devices in vivo. Indeed, the long-term presence of rigid electronics in vivo can cause infection, induce inflammation, and may even lead to fibrotic encapsulation, thereby enhancing the risk of implant rejection [167]. One avenue is the use of bioresorbable materials, which can be absorbed in vivo after completing their intended function. Both natural and synthetic polymers can be suitable candidates as bioresorbable electronics [168]. Ouyang et al. [185] have reported the fabrication of bioresorbable pressure sensors, which exhibited complete degradation in 63 to 84 d. Importantly, the use of similar materials for both vascular scaffolds and electronics can maintain the bioactivity of the scaffolds, which provides mechanical stability and biocompatibility with seamless integration. These scaffolds, when surface-functionalized with modifications, can remain unaffected by the incorporated electronics and maintain their antithrombotic and hemodynamic monitoring abilities [180]. Various types of electrically integrated vascular scaffolds, along with the mechanism of incorporated electronics in animal models, are listed in Table 4.

**Table 4.** Bioelectronic-integrated vascular scaffolds for monitoring hemodynamics, thrombosis, and restenosis

Systems	Materials	In vitro results	In vivo results	Working Mechanism	Ref
Battery-free wireless vascular platform	Inductive gold loops, polyimide connectors, and pressure sensors	Maintained signal integrity under balloon expansion at 14 atm	Implanted via catheter in the rabbit iliac artery; functional in blood and air environments	Wireless inductive coupling; capacitance-based pressure sensing	[173]
Piezoelectric 3-layered vascular graft	PVDF-Ag nanowire mat sandwiched between electrospun inner/outer PCL layers	Detect pulse and pressure; support endothelialization	Detected hemodynamic changes in a rabbit model	Piezoelectric deformation of PVDF converts pressure to voltage	[178]
Self-healing vascular graft with biosensor	Self-healing polymer, CNTs, and a lubricious top layer	Exhibit tunable mechanical properties; repellence to body fluids and proteins; prevent blood leakage	Anti-fouling and seamless integration in a rat model; maintain conductivity and structure after multiple needle punctures	Conductive sensor detects blood flow (10–100 ml/min) via resistance changes	[180]
Smart graphene-based vascular graft	Laser-induced graphene core with PDMS top/bottom layers	Thrombus detection and blood flow sensing	Detection of vascular blockage, thrombolytic drug response, and endothelialization	Graphene sensing layer measures resistance changes; wireless signal transmission	[181]
Flexible electronics wrapped around a bionic vascular conduit	PPy/PDMS encapsulated in PU/parylene C with AuNF electrode on backside	Detection of blood flow, pulse rate, stenosis, and thrombosis	Enhanced endothelialization and patency in rabbits; monitoring of blood flow and thrombosis in rabbits and monkeys	Triboelectric effect, where the contact–separation cycle generates voltage during vessel motion	[182]
Electronic-integrated AVG	Electrical impedance spectroscopy sensors and telemetry unit	Identified vascular cells and clot formation	Detection of blood clots and emboli in the swine carotid artery model	EIS detects impedance shifts from tissue and thrombus composition	[184]

PVDF-Ag, polyvinylidene fluoride-silver; PCL, polycaprolactone; CNTs, carbon nanotubes; PDMS, poly(dimethylsiloxane); PPy, polypyrrole; PU, polyurethane; AuNFs, gold nanofibers; AVGs, arteriovenous grafts; EIS, electrical impedance spectroscopy

## Challenges and Future Perspectives

Vascular scaffolds are essential tools for treating CVDs, yet their long-term performance is often compromised by early-stage thrombosis and late-stage IH formation. In this review, we introduced several leading elastomers applied for vascular scaffolds, summarized various antithrombotic and endothelium-inducing biofunctionalization strategies, and highlighted the integration of bioelectronics with vascular scaffolds for intelligent real-time monitoring of hemodynamics, thrombosis, and restenosis, which are valuable for supporting timely clinical intervention and improved post-operative management. Nevertheless, substantial challenges remain in translating these functionalized and smart scaffold systems into widespread clinical practice.

A wide range of elastomers has been explored for vascular scaffolds. In general, synthetic polymers offer advantages in mechanical properties, cost, and manufacturability, whereas natural materials endow excellent bioactivity. Therefore, combining these 2 classes of materials to harness their complementary strengths represents a promising strategy for engineering superior vascular scaffolds. Meanwhile, utilizing advanced manufacturing techniques, such as hybrid constructs, may achieve a hierarchical architecture that integrates suitable biomechanics, controllable degradation kinetics, and pro-regenerative capacity. Critically, the vascular scaffold must be multifunctional , which can be realized by incorporating anti-thrombogenic surfaces and promoting rapid endothelialization to prevent restenosis and ensure long-term patency.

However, current antithrombotic and endothelium-inducing modifications of vascular devices still face several challenges, including achieving uniform coating deposition, maintaining long-term stability under physiological flow, avoiding cytotoxic or immunogenic responses, and enabling cost-effective manufacturing [9,89,116]. Moreover, modifications designed to promote EC coverage may inadvertently stimulate VSMC proliferation, thereby increasing the risks of IH and ISR. It is therefore critical to strike a delicate balance between early antithrombotic effects and healthy re-endothelialization—without exacerbating SMC proliferation. Future research should prioritize the development of multifunctional and programmable modification systems to provide spatiotemporal control over biological responses, such as generating initial antithrombotic functionality followed by sustained pro-regenerative effects. Furthermore, the absence of standardized procedures also makes it difficult to compare the efficacy of different modifications, particularly when different experimental models or conditions are used across studies. For example, although promising results have been reported in small animal models, the transition to large animal models and eventual human trials is often hindered by the lack of robust, reproducible data.

Smart bioelectronic-integrated vascular devices already enable real-time monitoring of hemodynamics, thrombosis, and restenosis. However, additional functionalities—such as tracking mechanical remodeling and endothelialization progress on the scaffold—remain to be developed. Furthermore, compliance mismatch between implanted vascular grafts and native vessels can promote IH and subsequent graft failure [186]. Advancing high-performance monitoring of vascular grafts will require deeper insight into in vivo mechanobiological and hemodynamic interactions, underscoring the importance of the integration of biomechanical considerations into graft design [187]. Noninvasive and nondestructive monitoring approaches could also draw inspiration from advanced ultrasound-based techniques such as nonlinear resonant ultrasound spectroscopy (NRUS) and quantitative laser ultrasound visualization (QLUV), which are currently used in bone fragility assessment [188]. Notably, while existing bioelectronic-integrated vascular scaffolds can detect restenosis, thrombosis, and tissue overgrowth via mechanical, hemodynamic, and electrical signals, endothelial regeneration is still largely evaluated through indirect indicators. Variations in strain, pressure, impedance, and resistance have been associated with cellular responses and remodeling related to endothelialization [178,181,182,184]. However, the signal-based metrics do not directly measure endothelial coverage or functional maturation. Future bioelectronic vascular devices will therefore need endothelial-specific sensing strategies to enable direct, real-time evaluation of endothelial formation and phenotypic stability within vascular scaffolds.

For the clinical translation of smart electronic-integrated vascular scaffolds, the use of clinically approved materials is essential to ensure biocompatibility and implantability. Particularly, the development of compact wireless electronics for subcutaneous implantation and the integration of artificial intelligence for precise and remote vascular monitoring are indispensable [181]. Fully integrated intelligent vascular grafts can be fabricated by incorporating flexible electronics during the fabrication of vascular scaffolds, as exemplified by ferroelectric artificial arteries fabricated via 3D printing for real-time blood pressure monitoring and thrombosis detection, as well as electrospun nanofiber-based piezoelectric vascular grafts designed for mechanical sensing performance [178,189]. Further development requires an optimized structural design, including improvements in sensor encapsulation, sensor thickness, and the use of external reader electronics for long-term in vivo monitoring [173] as well as minimally invasive implantation techniques and strategies for data acquisition from bioelectronic devices. In parallel, nanogenerator-based sensors must achieve high energy density in ultrathin, lightweight architectures while minimizing mechanical mismatch with native tissues through modulus-matched materials and micro-/nanoscale structural design [190].

Looking ahead, next-generation vascular scaffolds are evolving toward more intelligent, multifunctional platforms that integrate thrombosis-resistant and endothelium-promoting biofunctionalization with real-time monitoring and adaptive regulation. By addressing the challenges in materials, biology, and engineering outlined above, such integrated systems hold the potential to fundamentally transform the management of CVDs, enabling more personalized, proactive, and precise therapeutic strategies.
